# Structator: fast index-based search for RNA sequence-structure patterns

**DOI:** 10.1186/1471-2105-12-214

**Published:** 2011-05-27

**Authors:** Fernando Meyer, Stefan Kurtz, Rolf Backofen, Sebastian Will, Michael Beckstette

**Affiliations:** 1 Center for Bioinformatics, University of Hamburg, Bundesstrasse 43, 20146 Hamburg, Germany; 2 Chair for Bioinformatics, University of Freiburg, Georges-Köhler-Allee 106, 79110 Freiburg, Germany; 3 Computer Science and Artificial Intelligence Lab, Massachusetts Institute of Technology, Cambridge, MA 02139, USA

## Abstract

**Background:**

The secondary structure of RNA molecules is intimately related to their function and often more conserved than the sequence. Hence, the important task of searching databases for RNAs requires to match sequence-structure patterns. Unfortunately, current tools for this task have, in the best case, a running time that is only linear in the size of sequence databases. Furthermore, established index data structures for fast sequence matching, like suffix trees or arrays, cannot benefit from the complementarity constraints introduced by the secondary structure of RNAs.

**Results:**

We present a novel method and readily applicable software for time efficient matching of RNA sequence-structure patterns in sequence databases. Our approach is based on affix arrays, a recently introduced index data structure, preprocessed from the target database. Affix arrays support bidirectional pattern search, which is required for efficiently handling the structural constraints of the pattern. Structural patterns like stem-loops can be matched inside out, such that the loop region is matched first and then the pairing bases on the boundaries are matched consecutively. This allows to exploit base pairing information for search space reduction and leads to an expected running time that is sublinear in the size of the sequence database. The incorporation of a new chaining approach in the search of RNA sequence-structure patterns enables the description of molecules folding into complex secondary structures with multiple ordered patterns. The chaining approach removes spurious matches from the set of intermediate results, in particular of patterns with little specificity. In benchmark experiments on the Rfam database, our method runs up to two orders of magnitude faster than previous methods.

**Conclusions:**

The presented method's sublinear expected running time makes it well suited for RNA sequence-structure pattern matching in large sequence databases. RNA molecules containing several stem-loop substructures can be described by multiple sequence-structure patterns and their matches are efficiently handled by a novel chaining method. Beyond our algorithmic contributions, we provide with *Structator *a complete and robust open-source software solution for index-based search of RNA sequence-structure patterns. The *Structator *software is available at http://www.zbh.uni-hamburg.de/Structator.

## Background

The discovery of new roles of non-coding RNAs (ncRNAs) has made them of central research interest in molecular biology [1,2]. Like proteins, ncRNA sequences that have evolved from a common ancestor can be grouped into families. For instance, the Rfam database [3,4] release 10.0 compiles 1,446 such families. Members of a family share, to different degrees, sequence and structure similarity. In many cases, however, the members of a family share only few sequence features, but share by far more specific structural and functional properties. Prominent examples of such cases are tRNAs and microRNA precursors.

In this paper, we consider the problem of searching nucleotide databases for occurrences of RNA family members. As sequence similarity is often remote even within well-established RNA families, we cannot rely on pure sequence alignment and related techniques for this task. Indeed, it has been shown that sequence alignments of structured RNAs fail at pairwise sequence identities below about 60% [5]. Therefore, we briefly review nucleotide database search methods that make use of sequence and structure information. There are general sequence-structure alignment tools, which determine structural similarities and derive consensus structure patterns for RNAs that are too diverse to be alignable at sequence level. We identify two classes of such tools. The first class, with *RNAforrester *[6] and *MARNA *[7] being the main representatives, require a known or predicted secondary structure for both sequences as input. However, they suffer from the low quality of secondary structure prediction, especially if the boundary of the RNA elements are not exactly known. The second class of methods are derivatives of the Sankoff algorithm [8], which provides a general solution to the problem of simultaneously computing an alignment and the common secondary structure of the two aligned sequences. Due to its high complexity (time and  memory) several variants of this approach have been introduced such as *foldalign *[9,10], *dynalign *[11] and *LocaRNA *[12]. Still, these tools have a time complexity that is generally too high for a rapid database search. Thus, more specialized tools for searching RNA families in nucleotide databases have been introduced. Tools like *RNAMotif *[13], *RNAMOT *[14], *RNABOB *[15], *RNAMST *[16], *PatScan *[17], and *PatSearch *[18] are based on motif descriptors defining primary and secondary structure properties of the families to be searched for. They provide a language for defining descriptors and a method to search with these in large nucleotide databases. For these tools, the motif descriptor for a family has to be extracted externally from other information (such as a multiple sequence-structure alignment) about the specific RNA family. There are also tools that automatically derive descriptors from structure-annotated sequences or a multiple sequence alignment of related RNA sequences such as *Infernal *[19,20], *RSEARCH *[21], and *PHMMTS *[22]. They use variants of stochastic context-free grammars as descriptors, whereas *ERPIN *[23] uses sequential and structural profiles. Despite being fast compared to other methods, descriptor-based tools available today have a running time that is, in the best case, linear in the size of the target sequence database. This makes their application challenging when it comes to large sequence databases. A solution with sublinear running time would require index data structures. However, widely used index structures like suffix trees [24] or arrays [25] or the FM-index [26] perform badly on typical RNA sequence-structure patterns, because they cannot take advantage of the RNA structure information. Here, we present a fast descriptor-based method and software for RNA sequence-structure pattern matching. The method consists of initially building an affix array [27], i.e. an index data structure of the target database. Affix arrays cope well with structural pattern constraints by allowing for an efficient matching order of the bases constituting the pattern. Structurally symmetric patterns like stem-loops can be matched inside out, such that first the loop region is matched and, in subsequent extensions, pairing positions on the boundaries are matched consecutively. Because the matched substring is extended to the left and to the right, this pattern matching scheme is known as bidirectional search. Unlike traditional left-to-right search where the two substrings constituting the stem region of the pattern are matched sequentially, in bidirectional search, base complementarity constraints are checked as early as possible. This leads to a significant reduction of the search space that has to be explored and in turn to a reduced running time. We note that bidirectional search for RNA sequence-structure patterns was also presented by Mauri et al. in [28]. However, their method uses affix trees [29] instead of the more memory efficient affix arrays. Affix trees require with approximately 45 bytes per input symbol more than twice the memory of affix arrays (18 bytes per input symbol), making their application infeasible on a large scale. Moreover, their method traverses the affix tree in a breadth-first manner, leading to a space requirement that grows exponentially with increasing reading depth. We instead employ a depth-first search algorithm whose space requirement is only proportional to the length of the searched substring.

The affix array directly supports the search for sequence-structure patterns that describe sequence-structure motifs with non-branching structure, for example stem-loops. In contrast, e.g. the search for stems closing a multi-loop is not directly supported. Nevertheless, even for RNA containing multi-loops, the affix array can still speed up the search. Our general approach for finding RNA families with branching structure is to describe each stem-loop substructure by a sequence-structure pattern. Each of these patterns is matched independently using the affix array. Then, with a new efficient chaining algorithm, we compute chains of matches such that the chained matches reflect the order of occurrence of the respective patterns in the molecule. Note that complex structures containing one or more multi-loops can be expected to contain sufficiently many non-branching patterns, such that the proposed chaining strategy identifies true matches with high specificity.

For a better understanding of the concepts underlying our method, we begin with formalizing RNA structural motifs. We then describe the concepts and ideas of affix arrays and show how to use them in an algorithm for fast bidirectional search for sequence-structure patterns. After presenting a detailed complexity analysis of the algorithm, we proceed with a detailed description and analysis of a novel method for computing chains of sequence-structure pattern matches. Finally, we benchmark and validate our method in several experiments.

## Methods

### Preliminaries

A *sequence S *of length *n *= |*S*| over an alphabet  is a juxtaposition of *n *elements (*characters*) from the set . *S*[*i*], 0 ≤ *i < n *denotes the *character of S at position i*. Let *ε *denote the empty sequence, the only sequence of length 0. By  we denote the set of sequences of length *n *≥ 0 over . The set of all possible sequences over  including the empty sequence *ε *is denoted by .

For a sequence *S *= *S*[0]*S*[1] ... *S*[*n *- 1] and 0 ≤ *i *≤ *j < n*, *S*[*i*..*j*] denotes the *substring S*[*i*]*S*[*i *+ 1] ... *S*[*j*] of *S*. We denote the *reverse sequence *of *S *with *S*^-1 ^= *S*[*n *- 1]*S*[*n *- 2] ... *S*[0]. For *S *= *uv*, *u *and , *u *is a *prefix *of *S*, and *v *is a *suffix *of *S*. The *k*-th suffix of *S *starts at position *k*, while the *k*-th prefix of *S *ends at *k*. Note that the 0-th suffix of *S *is *S *itself and that *S*[0] is the 0-th prefix of *S*. The *k*-th *reverse prefix *of *S *is the *k*-th suffix of *S*^-1^. For 0 ≤ *k *<*n*, *S_k _*denotes the *k*-th suffix of *S*, and , denotes the *k*-th reverse prefix of *S*.

Let  denote the *RNA alphabet *{*A*, *C*, *G*, *U*}. Its characters code for the nucleotides adenine (A), cytosine (C), guanine (G), and uracil (U). In the following we fix a sequence *S *over the RNA alphabet . For stating the space requirements of our index structures, we assume that |*S*|< 2^32^, such that sequence positions and lengths can be stored in 4 bytes.

### RNA structural motifs

**Figure 1:**
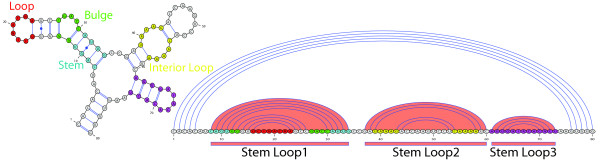
**Secondary structure elements of an RNA molecule represented by a base-pair graph (left) and as arc-annotated sequence (right)**. The depicted structure contains three stem-loop substructures. Observe that all arcs representing base pairings are *non-crossing *and stem-loop substructures can contain interior loops and bulges. Hence this molecule forms a *non-crossing *secondary structure that does not contain higher order structural elements like pseudoknots. Secondary structure drawings were generated with the *VARNA *program [55].

RNA molecules can form complex secondary structures consisting of different structural elements like stem-loops with or without bulges or internal loops. See Figure 1 for an overview of some secondary structure elements. Such elements are often important for the function of the molecule and are structurally conserved throughout evolution. The secondary structure is formed by Watson-Crick pairing of complementary bases and also by the slightly weaker wobble pairs. We say that two bases  are *complementary *and can form a *base pair *if and only if . A *non-crossing RNA structure R of length m *is a set of *base pairs *(*i*, *j*), 0 ≤ *i < j < m*, stating that the base at position *i *pairs with the base at position *j*, such that for all (*i*, *j*), (*i'*, *j'*) ∈ *R*: *i < i' *<*j' *<*j *or *i' *<*i < j < j' *or *i < j < i' *<*j' *or *i' *<*j' *<*i < j*. For the algorithms and methods presented in this paper we only consider this class of structures. For an example of such an RNA secondary structure see Figure 1. An important structural motif occurring in many RNA molecules is the *stem-loop *structure. We call *R *a *stem-loop *RNA structure if and only if for all (*i*, *j*), (*i'*, *j'*) ∈ *R *: *i < i' *<*j' *<*j *or *i' *<*i < j < j'*. Note that due to our definition a stem-loop can contain bulges and interior loops (see Figure 1). We equivalently call such a structure *non-branching*. In Figure 1, such stem-loop structures occur as substructures.

A *structure string H *is a sequence over the alphabet **{**., **(**,**)}** with an equal number of characters (and ). There is a bijection between the set of (non-crossing) RNA structures *R *and the set of structure strings *H*, both of length *m*, such that for each base pair (*i*, *j*) ∈ *R*, *H*[*i*] = (and *H*[*j*] = ), and *H*[*r*] = . for positions *r*, 0 ≤ *r < m*, that do not occur in any base pair of *R*, i.e. *r *≠ *i *∧ *r *≠ *j *for all (*i*, *j*) ∈ *R*. Due to this equivalence we identify both representations.

Let Φ = {R, Y, M, K, W, S, B, D, H, V, N} be a set of characters. The IUPAC nucleotide base code introduces the characters in Φ to code nucleotide ambiguity and assigns a specific character class  to each . In particular, for  and . A *sequence pattern *is a sequence . Let *m *denote its length |*P*|. An *occurrence *of *P *in a sequence *S *is a position *i*, 0 ≤ *i < n*, such that *P*[*k*] = *S*[*i *+ *k*] with *S*[*i *+ *k*] ∈ *φ*(*P*[*k*]) for all 0 ≤ *k < m*. An *RNA sequence-structure pattern (RSSP) * of length *m *is a pair of a *sequence pattern P *and a *structure string R*, both of length *m*. A *match *or *occurrence *of  of length *m *in an RNA sequence *S *is an occurrence *i *of *P *in *S*, such that for all base pairs (*l*, *r*) ∈ *R*: *S*[*i *+ *l*] and *S*[*i *+ *r*] are complementary. Furthermore, define  as a mapping of a character  to the set of its complementary characters in , i.e. .

In this paper, structures described by RSSPs are non-branching.

### The affix array data structure

In [27] the theoretical concept of an index data structure called *affix array *is described. This index structure supports efficient unidirectional as well as bidirectional searches and is more space efficient than the affix tree [29,30]. The term *unidirectional search *refers to the search for occurrences of a sequence pattern where the pattern characters are compared with sequence characters in a left-to-right (right-to-left) order, i.e. the already compared (matched) prefix (suffix), of the pattern is extended to the right (left). Notably, a change of the direction is not possible.

**Figure 2:**
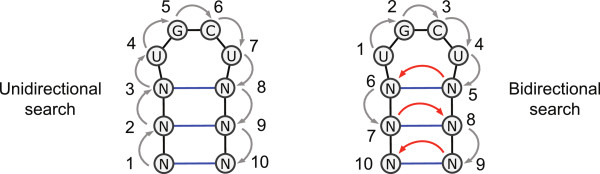
**Unidirectional (left) and bidirectional (right) searches for the RNA sequence-structure pattern (RSSP)  with *P *= NNNUGCUNNN and *R *= (((....))), which represents a stem-loop structure of length *m *= 10**. The numbers indicate the order in which the pattern characters are matched against the target sequence. In the unidirectional search, the characters are matched in a single direction, beginning (ending) with a character in *φ*(*P*[0]) (*φ*(*P*[*m *- 1])). In the bidirectional search, the loop region of the pattern can be matched first. Then, pairing bases are matched consecutively by switching the search direction, represented by the red arrows.

When searching for occurrences of sequence-structure patterns, however, unidirectional search cannot exploit the complementarity condition on base paired pattern positions. To utilize this condition as effectively as possible, both positions of a base pair need to be accessed immediately after each other. This is enabled by *bidirectional search*, which refers to methods where the direction of the match extension can be changed freely. Figure 2 illustrates the order of the character comparisons of a sequence-structure pattern in the unidirectional and bidirectional searches.

Until now, affix arrays have received little attention in bioinformatics. Presumably, this has been due to the lack of an open and robust implementation. As a consequence, their potential for efficient database search with RSSPs has hardly been recognized and the details of this data structure are not widely known in the field. Therefore, we briefly recall the basic ideas of the affix array, which constitutes the central component of our *Structator *approach.

For notational convenience, we define *S*^F ^= *S *and *S*^R ^= *S*^-1^. We use *S*^*X *^for statements that apply to *S*^F ^and *S*^R^. The subscript *X *is used for other notions depending on *S*^F ^and *S*^R ^in an analogous way. Furthermore, we introduce the notation  and . We reserve a character , called *terminator symbol*, for marking the end of a sequence. $ is lexicographically larger than all the characters in . The affix array data structure of a sequence *S *is composed of six tables, namely suf_F _and suf_R_, lcp_F _and lcp_R_, and aflk_F _and aflk_R_. They are called *suffix*, *longest common prefix*, and *affix link arrays *of *S*^F ^and *S*^R^, respectively. Table suf_R _is also known as *reverse prefix array*. suf*_X _*is an array of integers in the range 0 to *n *specifying the lexicographic order of the *n *+ 1 suffixes of the string *S*^*X*^$. That is,  is the sequence of suffixes of *S^X^*$ in ascending lexicographic order. Each of the tables suf_F _and suf_R _requires 4*n *bytes and can be constructed in  time and space [31]. In practice non-linear time [32,33] construction algorithms are often used as they are faster and require less space. lcp*_X _*is a table in the range 0 to *n *such that lcp*_X _*[0] = 0, and lcp*_X _*[*i*] is the length of the longest common prefix between  and  for 1 ≤ *i *≤ *n*. Each of the tables lcp_F _and lcp_R _requires *n *bytes and store entries with value up to 255, whereas occasional larger entries are stored in an exception table using 8 bytes per entry [34]. More space efficient representations of the lcp table are possible (see [35]). The construction of lcp_F _and lcp_R _can be accomplished in  time and space given suf_F _and suf_R _[36]. In contrast to [27] where affix arrays were described using a terminology derived from tree-like data structures, we explain the underlying concepts of this data structure in terms of intervals in the suffix array suf*_X _*. Two important concepts of affix arrays are suffix-intervals and lcp-intervals. An interval [*i*..*j*] representing the set of suffixes , 0 ≤ *i *≤ *j *≤ *n*, of *width j *- *i *+ 1, is a *suffix-interval *in suf*_X _*with *depth (prefix length) ℓ *∈ {0,..., *n*}, or *ℓ-suffix-interval*, denoted *ℓ *- [*i*..*j*], if and only if the following three conditions hold:

1. lcp*_X _*[*i*] <*ℓ*;

2. lcp*_X _*[*j *+ 1] <*ℓ*; and

3. lcp*_X _*[*k*] ≥ *ℓ *for all *k *∈ {*i *+ 1,..., *j*}.

We call a suffix-interval *ℓ *- [*i*..*j*] in suf_*X *_*lcp-interval *in suf*_X _*with *lcp-value ℓ *∈ {0,..., *n*}, or *ℓ-interval*, if and only if *i < j *and lcp*_X _*[*k*] = *ℓ *for at least one *k *∈ {*i *+ 1,..., *j*}.

For a suffix-interval *ℓ *- [*i*..*j*] in suf*_X _*, we denote the common prefix of length *ℓ *of its suffixes  by *δ_X_*(*ℓ *- [*i*..*j*]) = *S^X^*[suf*_X _*[*i*]..suf*_X _*[*i*] + *ℓ *- 1]. In case of an lcp-interval *ℓ *- [*i*..*j*] in suf*_X _*, *δ_X _*(*ℓ *- [*i*..*j*]) is the longest common prefix of all suffixes in this interval.

**Figure 3:**
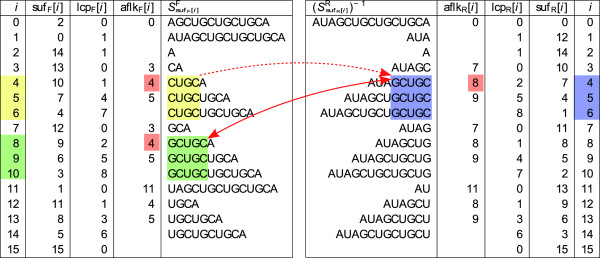
**Affix array for *S *= AUAGCUGCUGCUGCA**. Some lcp-intervals are marked by rectangles and the affix links from an lcp-interval to its reverse interval are represented by arcs. The solid arc points in two directions, from the the lcp-interval *q *= 5 - [8..10] in suf_F _(on the left-hand side) to its reverse interval *q*^-1 ^= 5 - [4..6] in suf_R _(on the right-hand side) and vice versa. That is, *q *= (*q*^-1^)^-1 ^(see Lemma 2). The dotted arc points in only one direction, from the lcp-interval *q *= 4 - [4..6] in suf_F _to its reverse interval *q*^-1 ^= 5 - [4..6] in suf_R_. In this case, the reverse of *q*^-1 ^is (*q*^-1^)^-1 ^= 5 - [8..10], and *q ≠ *(*q*^-1^)^-1^.

In summary, a suffix-interval *ℓ *- [*i*..*j*] in suf*_X _*describes simultaneously:

• A location in the index structure suf*_X _*by interval borders *i *and *j *and depth *ℓ*. For an example, see the yellow marked region in Figure 3 which corresponds to the suffix-interval 4 - [4..6] in suf_F_.

• A (lexicographically ordered) sequence of suffixes . For an example, consider the lexicographically ordered sequence  of suffixes in the suffix-interval 4 - [4..6] in suf_F _in Figure 3.

• A substring of *S*^*X *^of length *ℓ*, namely *δ_X_*(*ℓ *- [*i*..*j*]). That is, for the suffix-interval 4 - [4..6] in suf_F _in Figure 3, *δ*_F_(4 - [4..6]) = CUGC.

• The occurrences of this substring in *S^X^*, namely at positions suf*_X _*[*i*],..., suf*_X _*[*j*]. To give an example, consider Figure 3 and observe that substring CUGC occurs at positions suf_F_ [4] = 10, suf_F_ [5] = 7, and suf_F_ [6] = 4 in *S*^F ^= AUAGCUGCUGCUGCA.

For unidirectional left-to-right search of some pattern in *S *it is sufficient to process lcp-intervals only in suf_F_. For bidirectional pattern search using affix arrays, described in detail in the next section, we employ information from table suf_F _as well as suf_R_. Therefore, we need to associate information of one table to the other. This is done by linking intervals via tables aflk_F _and aflk_R_. We observe that there exists a mapping between lcp-intervals in suf_F _and suf_R_. This is stated by the following proven lemma [27].

**Lemma 1 ***For every lcp-interval q *= *ℓ *- [*i*..*j*] *in table *suf_*X *_*there is exactly one lcp-interval q*^-1 ^= *ℓ' *- [*i'*..*j'*] *in table ** called reverse lcp-interval of q, such that ℓ' *≥ *ℓ and the ℓ *- 1*-th prefix of ** equals *(*δ*_*X*_(*q*))^-1^. *The number of suffixes (prefixes) represented by q and q*^-1 ^*are the same*, *i*.*e*., *j *- *i *= *j' *- *i'*.

We note that the equivalence *q *= (*q*^-1^)^-1 ^is not necessarily true. This is stated by the next lemma.

**Lemma 2 ***If the lcp-interval q*^-1 ^*with depth ℓ' in ** is the reverse of the lcp-interval q with depth ℓ in *suf*_X _and ℓ *= *ℓ'*, *then q *= (*q*^-1^)^-1^. *Otherwise*, *if ℓ' > ℓ*, *then q *≠ (*q*^-1^)^-1^.

The mapping between intervals in *S*^F ^and *S*^R ^is encoded in tables aflk_F _and aflk_R _as follows. Tables aflk_F _and aflk_R _store, for each lcp-interval in suf_F _and suf_R _respectively, a pointer to the reverse interval in the reverse tables  and . The position in the tables where the pointers are stored is determined by the function home*_X _*, defined as(1)

where *ℓ *- [*i*..*j*] is an lcp-interval in suf*_X _*. Hence, the home position is one of two boundary positions. Strothmann [27] shows that home*_X _*([*i*..*j*]) ≠ home*_X _*([*i'*..*j'*]) for different lcp-intervals *ℓ *- [*i*..*j*] and *ℓ' *- [*i'*..*j'*].

Table aflk*_X _*of string *S^X^*$ with total length *n *+ 1 can now be defined as a table in the range 0 to *n *such that aflk*_X _*[home*_X _*(*q*)] = *i'*, where *q *is an lcp-interval in suf*_X _*and *i' *is the left border of the reverse interval *q*^-1 ^= [*i'*..*j'*] in . We refer to the entries in table aflk*_X _*as *affix links*. Tables aflk_F _and aflk_R _occupy 4*n *bytes each. They can be computed by traversing the lcp-intervals in suf*_X _*while simultaneously looking for the corresponding reverse lcp-intervals in . Locating reverse lcp-intervals can be accelerated by skp-tables. These tables, introduced in Beckstette *et al. *[37] and hereinafter referred to as skp_F _and skp_R_, can be constructed in linear time [38] and allow one to quickly skip intervals in suf*_X _*(for details, see [37]).

The construction of tables aflk_F _and aflk_R _takes  time. Although the use of skp-tables requires additional 2 × 4*n *bytes of memory, they considerably reduce the construction times of tables aflk_R _and aflk_R _in practice. We note that Strothmann [27] describes a linear time construction algorithm for tables aflk_F _and aflk_R_, which employs suffix link and child-tables [34] and an additional table. Altogether these tables require together at least additional 7*n *bytes of space. Moreover, even without applying the skp-table based acceleration, Strothmann states that the quadratic time construction algorithm is fast in practice. An example of the affix array for sequence *S *= AUAGCUGCUGCUGCA highlighted with some of its lcp-intervals connected to the respective reverse interval via the aflk*_X _*table is shown in Figure 3.

Because affix links in table aflk*_X _*are only defined for lcp-intervals but not suffix-intervals in general, which we require in bidirectional search, we introduce the concept of *affix-intervals*. Affix-intervals are similar to affix nodes as defined in [27]. An affix-interval in suf*_X _*is a triple *v *= 〈*k*, *q*, *X*〉, where *k *is an integer designated *context *of *v *and *q *is a suffix-interval in suf*_X _*.

An affix-interval *v *= 〈*k*, *q*, *X*〉 in suf*_X _*, with *q *= *ℓ *- [*i*..*j*], *ℓ >*0, -*m < k < ℓ*, describes a substring *ω_X_*(*v*) of *S^X ^*of length *ℓ *- *k*, defined as the *k*-th suffix of *δ_X_*(*q*), i.e. *ω_X_*(*v*) = *S^X^*[suf*_X _*[*i*] + *k*..suf*_X _*[*i*] + *ℓ *- 1]. At the same time *v *identifies all occurrences of *ω_X_*(*v*) in *S^X^*, namely the positions suf*_X _*[*i*] + *k*,..., suf*_X _*[*j*] + *k*.

For *v * = 〈*k*, *q*, *X*〉, we therefore also use the notation  if *X *= F and  if *X *= R. As an example, consider the affix-interval *v *= 〈1, 4 - [4..6], F〉 in suf_F _of the affix array shown in Figure 3. In this case, *k *= 1, *q *= 4 - [4..6], and *X *= F. *v *identifies all occurrences of substring  in *S*^F ^at positions suf_F_ [4] + 1 = 11, suf_F_ [5] + 1 = 8, and suf_F_ [6] + 1 = 5. Observe that  is the first suffix of *δ*_F_(*q*) = CUGC due to context *k *= 1.

### Searching RNA databases for RSSPs with affix arrays

Pattern matching using affix arrays means the sequential processing of characters in the pattern guiding the traversal of the data structure. This can be performed in either a traditional left-to-right order resulting in a unidirectional search or in a bidirectional way where character comparison is started at any position of the pattern extending the already matched substring of the pattern to the left or to the right. We will see that bidirectional search using alternating series of left and right extensions is very well suited for fast database search with RNA sequence-structure patterns (RSSPs) containing both paired and unpaired bases. In the following we will explain the two different traversal strategies underlying unidirectional and bidirectional search using affix arrays.

#### Unidirectional traversal

**Figure 4:**
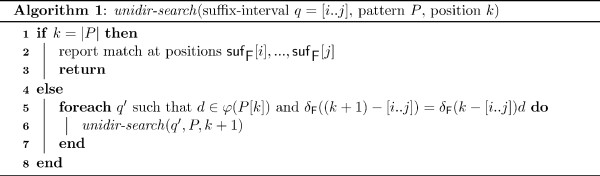
**Unidirectional search algorithm for searching for a sequence pattern **. Given the suffix array suf_F _of *S*, the procedure enumerates all occurrences of *P *in *S *when called by *unidir-search*([0..|*S*|], *P*, 0). In line 5, the suffix-interval *q' *is located by binary search in .

Let  be a sequence pattern to be searched in *S *in a unidirectional left-to-right way using information from table suf_F _only. To search for *P *, we call the procedure *unidir-search *of Figure 4 by *unidir-search*([0..|*S*|], *P*, 0). Therefore, in step 0 we start searching for the characters in *φ*(*P*[0]) in the suffix-interval *q*_0 _= 0 - [0..*n*] in suf_F_, which represents all suffixes of *S*$. In each step *k*, *k *≥ 0, we locate the *k *+ 1-suffix-intervals *q_k _*of maximal width, such that *P *[0..*k *- 1]*d *matches *δ*_F_(*q_k_*). For each *d *∈ *φ*(*P *[*k*]), this step is performed by a binary search in the suffix-interval *q*_*k*-1 _= *ℓ *- [*i*..*j*] for *q_k _*= (*ℓ *+ 1) - [*i'*..*j'*], *i *≤ *i' *≤ *j' *≤ *j*, *j' *- *i' *maximal, and *S*[suf_F_[*i'*] + *k*] = *d*.

After *m *steps, if all *q_k _*could be located, *δ*_F_(*q_m_*), *q_m _*= *m *- [*r*..*s*], matches the pattern *P *and the occurrences suf_F_[*r*], suf_F_[*r *+ 1],..., suf_F_[*s*] of *δ*_F_(*q_m_*) are reported as occurrences of *P *in *S*. Note that in this approach the matched substring of *S *is extended only to the right and at each step *k *the occurrences of the already matched prefix are represented by a suffix-interval.

#### Bidirectional traversal

For the bidirectional search, we start at some position in  and then compare the pattern *P *character by character to the text, where we can freely switch between extending to the left or to the right. Note that as in the case of unidirectional search, ambiguous nucleotides *x *in the pattern can be handled by enumerating all characters *c *in the corresponding character class *φ*(*x*). We can focus on the situation in the search, where

• a range *r*..*r' *(0 ≤ *r *≤ *r' *<*m*) of the pattern *P *is already compared,

• the occurrences of a substring  of *S *matching *P*[*r*..*r'*] are represented by an affix-interval *v *= 〈*k*, *ℓ *- [*i*..*j*], *X*〉 in suf*_X _*, and

• we want to extend  either to the left or to the right by a sequence character  (that matches the respective pattern character *P*[*r *- 1] or *P*[*r' *+ 1]). This will result in a new, extended affix-interval *v_x_*.

##### Switch of the search direction

Like its suffix-interval, an affix-interval directly supports extension of the represented substring in only one direction, namely searching to the left for *X *= F and to the right for *X *= R. However, there are "corresponding" affix-intervals representing the same substring of *S *but allowing extension to the opposite direction.

If the new search direction differs from the supported search direction of *v*, this *switch of the search direction *requires determining the corresponding affix-interval *v' *in  unless *i *= *j *or *v *has non-empty context *k *≠ 0. There are these two exceptions, since first if *i *= *j*, independently of the value of *k*, *ω_X_*(*v*) is already a unique substring of *S^X^*. Second, for a non-empty context *k *≠ 0, all occurrences of substring *ω_X_*(*v*) in *S*^*X *^are followed (if *k *> 0) or preceded (if *k *< 0) by the same substring .

Let *k *= 0 and *i < j*. The affix-interval  in  is called the *reverse affix-interval *of *v *= 〈*k*, *ℓ *- [*i*..*j*], *X*〉 if and only if *j' *- *i' *= *j *- *i*, *ℓ' *≥ *ℓ*, and . The interval boundaries *i' *and *j' *of *v' *are determined via a lookup in table aflk*_X _*. We set *i' *= aflk*_X _*[home*_X _*([*i*..*j*])] and *j' *= *i' *+ (*j *- *i*). Observe that *ℓ *is not necessarily the length of the longest common prefix of all suffixes in [*i*..*j*]. For this reason we define *ℓ*_lcp _= *min*{lcp*_X _*[*k*] | *i < k *≤ *j*} ≥ *ℓ *and compute the context of *v' *as *k' *= *ℓ*_lcp _- *ℓ*. Further, we set *ℓ' *= *ℓ*_lcp_. Hence the reverse affix-interval  is well defined and *v' *is the required corresponding interval of *v*.

##### Right/left *c*-extension of an affix-interval

In our situation,  represents the occurrences of a substring *u *of *S *matching *P*[*r*..*r'*].

The *right (left) extension of v by a character *, also called *c-extension of v*, is an operation that computes the affix-interval *v_x _*representing all occurrences of a substring *uc *(*cu*). It fails, if there is no such substring. We elaborate the cases for right extension. The cases for left extension are symmetric and therefore omitted. For right *c*-extension of *v *= 〈*k*, *ℓ *- [*i*..*j*], *X*〉, we determine the interval *v_x _*= 〈*k_x_*, *ℓ_x _*- [*i_x_*..*j_x_*], *X_x_*〉 with . The first two cases do not require switching the search direction.

• Case *X *= F and *i *= *j*. *u *is a unique substring  of *S*. If *S*[suf_F_[*i*] + *ℓ*] = *c*, then *v_x _*= 〈*k*, (*ℓ *+ 1) - [*i*..*j*], F〉.

• Case *X *= F and *i < j*. We determine the minimal *i_x _*≥ *i *and maximal *j_x _*≤ *j *in suf_F _such that *S*[suf_F_[*i_x_*] + *ℓ*] = *c *and *S*[suf_F_[*j_x_*] + *ℓ*] = *c *by binary search in the suffix-interval *ℓ *- [*i*..*j*]. If *i_x _*and *j_x _*exist, we set *v_x _*= 〈*k*, (*ℓ *+ 1) - [*i_x_*..*j_x_*], F〉.

The following cases require switching the search direction.

• Case *X *= R, *i *= *j*. We evaluate *S*^R^[suf_R_[*i*] + *k *- 1]. If *S*^R^[suf_R_[*i*] + *k *- 1] = *c*, set *v_x _*= 〈*k *- 1, *ℓ *- [*i*..*j*], R〉.

• Case *X *= R, *i < j*, and *k *= 0. We first determine the reverse affix-interval *v' *= 〈*k'*, *ℓ' *- [*i'*..*j'*], F〉 of *v *via a switch of the search direction as described above. Then we compute the minimal *i_x _*≥ *i' *and maximal *j_x _*≤ *j' *via binary search, such that *S*[suf_F_[*i_x_*] + *ℓ'*] = *c *and *S*[suf_F_[*j_x_*] + *ℓ'*] = *c*. If *i_x _*and *j_x _*exist, we set *v_x _*= 〈*k'*, (*ℓ' *+ 1) - [*i_x_*..*j_x_*], F〉.

• Case *X *= R, *i < j*, and *k >*0. We evaluate the (*k *- 1)-th character of *δ*_R_(*ℓ *- [*i*..*j*]). That is, if *δ*_R_(*ℓ *- [*i*..*j*])[*k *- 1] = *c*, then we consume the context *k *by setting *v_x _*= 〈*k *- 1, *ℓ *- [*i*..*j*], R〉.

The operation fails if *v_x _*cannot be determined.

#### RSSP matching using affix arrays

Searching a sequence *S *with an RNA sequence-structure pattern (RSSP)  means to find the occurrences of *P *in *S *under the complementarity constraints imposed by the structure string *R *(cf. our definition of RSSP-occurrence). We introduce a search algorithm that checks for complementarity constraints as early as possible in bidirectional search to maximally reduce the search time due to this restriction.

For further considerations, we will assume a special 'canonical' form for RSSPs, which we define in the following. Independently of a sequence *S*, each RSSP describes a set of pattern instances, i.e. the set of potential subsequences matching the pattern. Often, there are several patterns that describe the same set of instances. For example, the pattern (UNUACACGNR, ( ( ( . . . . ) ) ) ) describes the same set of instances as (UNUACACGNR, ( ( . . . . . . ) ) )  since the additional base pair (2, 7) in ( ( ( . . . . ) ) ) does not make the pattern more specific. We will define a pattern to be structure minimal if there is no, in this sense, equivalent pattern containing a true subset of the base pairs. An RSSP  is *structure minimal *if and only if for all base pairs (*i*, *j*) ∈ *R *it holds that

Furthermore, a general pattern is called *inconsistent *if it does not have any instance. Formally, a pattern is *consistent *if and only if for each base pair (*i, j*) it holds that  and . An example of an inconsistent RSSP is  with *P *= UAUACACGAN and *R *= ( ( . . . . . . ) ). is not consistent because there is a base pair (1, 8) ∈ *R *but the bases *P*[1] = A and *P*[8] = A are not complementary. An example of a structure minimal and consistent RSSP is (UNUACACGNR, ( ( . . . . . . ) ) ). Note that a pattern can be transformed into an equivalent structure minimal pattern and checked for consistency in  time. For complexity considerations, we can therefore safely assume that patterns are consistent and structure minimal.

In this case, one can restrict the search space by comparing the two positions of each base pair immediately after each other. Due to this, the enumeration of characters matching the pattern symbols at each base pair can be restricted to the smaller number of complementary ones. In the search for a sequence-structure pattern this can reduce the number of enumerated combinations of matching characters exponentially. Thus, for structure minimal patterns (*P, R*), the non-branching structure *R *suggests a search strategy, i.e. an order of left and right extensions, which requires switching the search direction at every base pair but makes optimal use of the complementarity constraints due to the base pairs.

**Figure 5:**
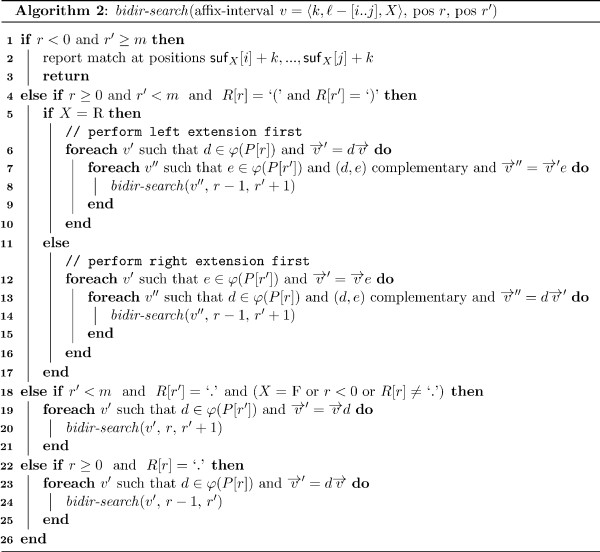
**Bidirectional recursive RSSP matching using an affix array**. Procedure *bidir-search *finds all matches of a given RSSP (*P, R*), beginning the pattern extensions from any position in the loop region or any position in a completely unpaired pattern. In each call, parameter *v *denotes the affix-interval representing matches of the pattern substring *P*[*r *+ 1..*r' *- 1], 0 ≤ *r *≤ *r' *<*m *satisfying the structural constraints imposed by *R*[*r *+ 1..*r' *- 1]. The procedure takes care to change the search direction only as often as necessary, in particular it changes the direction only once per base pair.

Following this idea, Mauri and Pavesi [28] presented an algorithm for matching RNA stem-loop structures using affix trees. This algorithm explores the search space in a breadth-first manner, so memory use grows exponentially with increasing depth. Instead of an affix tree, we employ the more space efficient affix array data structure and use a depth-first search algorithm which only requires space for the search proportional to the length of the substring searched. The depth-first search for all occurrences of a stem-loop RSSP  is performed by calling procedure *bidir-search *of Algorithm 2 (see Figure 5). Note that we explicitly support bulges and internal loops in the stem-loop pattern, i.e. we do not require perfect stacking of the base pairs but allow general non-branching structures.

In our algorithm, we switch the search direction only once per base pair when matching the stem region of the pattern, thus halving the number of lookups in the affix link tables compared to a naive algorithm without this optimization. This was also observed by Strothmann [27] whose algorithm did not support RSSPs containing bulges and internal loops.

To match  we call procedure *bidir-search *initially as *bidir-search*(〈0, 0 - [0..*n*], F〉, *r*_0 _- 1, *r*_0_), where 〈0, 0 - [0..*n*], F〉 is an affix-interval and *r*_0 _is any position in the loop region of the RSSP or any position of a completely unpaired pattern. Then, the procedure traverses the affix-intervals by performing right and left extensions, while at the same time checking base complementarity of paired positions. This verification takes constant time by using a binary table of size  containing all valid base pairings. Matching positions are reported whenever the boundaries of the RSSP are reached.

In principle, we are free to choose any loop position *r*_0 _(or any position if *R *is empty) for starting our bidirectional search algorithm. However, in order to reduce the combinatorial explosion of the search space due to ambiguous IUPAC characters, it is preferable to match non-ambiguous pattern characters first. To keep the selection simple, we set *r*_0 _to the position of the first character *c *in the possible range such that |*φ*(*c*)| is minimal. That is, we start the search with the most specific (least ambiguous) character.

A detailed example of bidirectional RSSP search along with the underlying affix array traversal is provided in Additional file 1 Section S1. We remark that procedure *bidir-search *can be extended to support variable-length RSSPs. Such an extended version of *bidir-search *is provided in Additional file 1 Section S3.

#### Analysis

We analyze the complexity for searching in a sequence *S *of length *n *for an RSSP  of length *m < n*, where the index structures for *S *are already computed.

The bidirectional search algorithm requires tables suf_F _and suf_R_, lcp_F _and lcp_R_, and aflk_F _and aflk_R_. Under our assumption that *n <*2^32^, each of the four tables suf*_X _*and aflk*_X _*consumes 4*n *bytes, and the two tables lcp_X _are each stored in *n *bytes (*X *∈ {F, R}). This amounts to a space consumption of 18*n *bytes for the index structures. The algorithm performs a depth first search, where the depth is limited by *m*, and therefore requires  space. The total space complexity is therefore .

We assume that  is structure minimal. Such a pattern  without ambiguity, i.e. , does not contain base pairs and the search for  does not profit from bidirectional search. Although such a pattern is processed by Algorithm 2, it can be handled by Algorithm 1 using only a suffix array and saving some overhead.

Algorithm 1 accomplishes the search for a non-ambiguous pattern  on the suffix array suf_F _using binary search for locating intervals in  time, where *z *is the number of occurrences of *P *in *S*. We remark that this time bound can be lowered at the price of higher memory consumption to [25] or even [34,39] time by using additional precomputed information.

Notably, if there is ambiguity but no base pair in , bidirectional search can still be beneficial in practice. This is the case when searching for a pattern in which a string of unambiguous characters is surrounded on both sides by ambiguous IUPAC characters, because the comparison can start at the most specific part of the pattern. The time complexities for searching ambiguous patterns with Algorithm 1 can be estimated as  in the worst case of searching for the sequence pattern *P *consisting only of Ns. Furthermore, note that our Algorithm 2 behaves exactly like Algorithm 1 on patterns without base pairs if we invoke the search procedure with *r *= -1 and *r' *= 0.

For a pattern  of length *m*, let *p *≥ 0 be the number of base pairs in *R*. In the worst case *P *consists only of Ns. Moreover, all possible strings of length *m *satisfying the complementarity constraints specified in *R *occur in the text *S*. Recall that, since we allow (G, U) pairs, there are  possible complementary base pairs. Thus, there are  such strings and Algorithm 2 spans a virtual tree with  paths from the root to a leaf. At each leaf, it reports the occurrences of the respective matched substring.

On each path from the root to the leaf the algorithm performs *m *- 2*p c*-extensions and at most one switch of the search direction for matching the *m *- 2*p *unpaired characters. Then, it performs 2*p *c-extensions and *p *switches of the direction for matching the base paired positions. Therefore, we count the total number of c-extensions as

which is in .

The cost of each *c*-extension consists of the cost of locating the suffix-interval of the new affix-interval, which is performed by binary search in , and the cost for potentially computing the reverse affix-interval when switching the search direction.

Instead of performing the binary search over the suffix tables, one can use the child-tables introduced by Abouelhoda *et al*. in [34] to determine the child intervals and switch the search direction in constant time. The child-tables, however, add at least 2*n *bytes to the index and require additional involved index construction. As the child-tables improve the worst case behavior but, on the other hand, require more space, we analyze the complexity with and without these tables (i.e. with tables suf*_X_*, lcp*_X_*, and aflk*_X _*only).

First, we analyze the time required for performing a single switch of the search direction. Therefore we assume that the current affix-interval is *v *= 〈*k*, *ℓ *- [*i*..*j*], *X*〉. Consider the following two cases.

(1) Case *i *= *j *or *k ≠ *0. If *i *= *j*,  represents a unique substring of *S*, or, if *k ≠ *0, all occurrences of  substring  in *S *are followed (if *k >*0) or preceded (if *k <*0) by the same substring of length |*k*| (known as context). Switching the search direction does not require locating the reverse interval of *v*, because the algorithm can perform the *c*-extension in the new search direction by consuming context. Therefore, this case requires constant time.

(2) Case *i < j *and *k *= 0. The algorithm needs to locate the reverse affix-interval  of *v*. Interval boundaries *i' *= aflk*_X _*[home*_X _*([*i..j*])] and *j' *= *i' *+ (*j *- *i*) of *v' *are computed in constant time.

By definition, computing the reverse affix-interval of *v *requires knowing *ℓ*_lcp_. Then, *ℓ' *= *ℓ*_lcp _and *k' *= *ℓ' *- *ℓ*. Without child-tables, we determine *ℓ*_lcp _by computing the length of the longest common prefix between  and . It suffices to perform *ℓ*_lcp _- *ℓ *+ 1 = *k' *+ 1 character comparisons only, since both suffixes  and  share a common prefix of at least length *ℓ*. With the help of child-tables, *ℓ*_lcp _is determined in constant time [34].

Due to the following lemma, the computation of all reverse affix-intervals on one path of our virtual tree is in  if child-tables are not used.

**Lemma 3 ***Using tables *suf*_X_*, lcp*_X_, and *aflk*_X_, the computation of all contexts on a path in the recursion of Algorithm 2 is in *.

**Proof**. Let *v*_1_, *v*_2_, *v*_*t *_..., *v*_*C *_be the sequence of reverse intervals processed when matching , and let *k_t _*denote the context of *v_t _*for 1 ≤ *t *≤ *C*.

To show , let *v *= 〈*k*, *ℓ *- [*i..j*], *X*〉, with *k *= 0, *i < j*, and *X *= F (*X *= R), be the current affix-interval. We assume without loss of generality that we perform a left (right) *c*-extension of *v *and thus locate the reverse interval . Then the following statements hold: *k_t _*≥ 0, *ℓ_t _*= *ℓ *+ *k_t_*, and *j_t _*- *i_t _*= *j *- *i *(see Lemma 1). Observe that *k_t _*= 0 implies  and *k*_*t *_> 0 implies that substring  has a non-empty prefix of length *k_t_*, namely .

Note that *v_t _*is only located if *k *= 0, otherwise the context *k *has to be consumed. Hence there is no reverse interval , with 1 ≤ *s *≤ *C*, *s *≠ *t*, and *k*_*s *_> 0, such that the (*k_s _*- 1)-th prefix of  overlaps with  for the same positions in . From this,  follows. Since a single context *k_t _*can be determined by performing exactly *k_t _*+ 1 character comparisons, this implies  time to compute all these contexts. With this, we conclude that all switches of the search direction performed while finding one substring *w *in *S *that matches  take up to  time. □

Therefore, when searching for  without child-tables, the total time for switching search directions is coarsely estimated by multiplying the complexity for one path with the number of paths as . The use of child-tables removes the linear factor.

For the worst case that all strings matching the pattern actually occur as substrings in *S*, the sequence *S *must have a certain minimal length. In the case of *p *= 0, the possible matches are the words in  and a sequence that contains all these matches is called -ary *de Bruijn *sequence of order *m *[40] without wrap-around, i.e. a *de Bruijn *sequence with its first *m *- 1 characters concatenated to its end. Such a sequence was shown to have a length of . As a consequence, the worst case requires *n *≥ *n*_0_.

We summarize the worst-case time complexities for Algorithm 2 as follows. 1.) From determining new suffix-intervals, we get a contribution of . For *n *≥ *n*_0_, this is in . Child-tables reduce this time further to . 2.) Switching directions without child-tables is in  worst-case time, which is reduced to  when using child-tables. For *n *≥ *n*_0_, *E_m, p _*is in . Finally, Algorithm 2 runs in , which is reduced to  using child-tables (i.e.  for *n *≥ *n*_0_).

One should note that the worst-case time complexity of bidirectional search for sequence-structure pattern is only in the order of online search algorithms. In our implementation, we use a minimal set of tables in order to keep the implementation simple and save space.

However, it can be clearly seen from this analysis that the worst case is based on extremely pessimistic assumptions that are almost contrary to the expected application. 1.) It is assumed that a pattern consists of wildcards N only. In the expected application, however, patterns will often specify bases in the loop region, which is of particular benefit for our algorithm. 2.) Sequences, like the *de Bruijn *sequence, that contain all possible matches of an average sized pattern will be rare in practice. E.g. it could be assumed that a sequence that contains all possible matches of a pattern *Q *with *p *base pairs (and *P *= N ... N) is at least as long as the -ary *de Bruijn *sequence of order *m*, since one expects no significant bias for the specific complementarity due to *R *over all substrings of length *m*. However,  is even for small *p *much smaller than *n*_0 _= 4*^m ^*+ *m *- 1. For example, four base pairs (i.e., *p *= 4) reduce the time bound by a factor of (16*/*6)^4 ^≈ 50 and eight base pairs reduce time by a factor of about 2500.

### RNA secondary structure descriptors based on multiple ordered RSSPs

Obviously RNAs with complex, branching structures cannot be described completely by a single RSSP. Describing an RNA by only a single unbranched fragment is often inappropriate, since searching a large sequence database or a complete genome for structurally conserved RNAs (RNA homology search) with a single RSSP will likely generate many spurious matches. However, larger RNAs can often adequately be described by a sequence of RSSPs. This holds for 1,247 out of 1,446 RNA families in Rfam 10.0 which have a structure containing several stem-loops but no multi-loop. Only 199 out of 1,446 (13.76%) RNA families in Rfam 10.0 containing multi-loops cannot be modeled completely this way. Still, the consensus structures of these 199 families contain on average 4.06 stem-loops (standard deviation 2.08, median 3) which can be modeled as RSSPs. In consequence, we can use a sequence of RSSPs that consist of at least one pattern per stem-loop (and potentially also unstructured patterns) for the description of those families. This allows to accurately identify members even of those families containing multi-loops.

**Figure 6:**
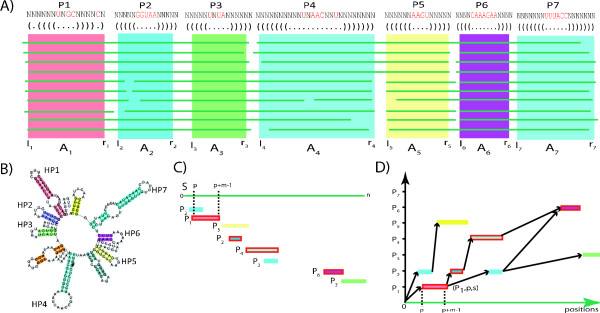
**Construction of RNA secondary structure descriptors. **(A) Non-overlapping alignment blocks of stem-loop regions excised from a multiple sequence-structure alignment and derived sequence-structure patterns. Since *l_i _*≤ *r*_*i *_<*l*_*j *_≤ *r_j _*and sequence regions *S*[*l*_*i *_... *r*_*i*_] fold into stem-loop structures for 1 ≤ *i *≤ *j *≤ 7, *A *= *A*_1_, *A*_2_, *A*_3_, *A*_4_, *A*_5_, *A*_6_, *A*_7 _is an ordered sequence of non-overlapping alignment blocks suitable to construct an RNA secondary structure descriptor . The sequence-structure patterns , *i *∈ [1, 7] of  given on top of their underlying alignment blocks describe the seven marked stem-loops shown in the RNA secondary structure (B) of the Citrus tristeza virus replication signal (Rfam: RF00193). (C) Matches of RSSPs , *i *∈ [1, 7], on sequence *S*, sorted in ascending order of their start position. (D) Graph-based representation of the matches of , *i *∈ [1, 7]. An optimal chain of collinear non-overlapping matches is determined by computing an optimal path in the directed acyclic graph. Observe that not all edges in the graph are shown in this example and that the optimal chain (indicated here by their red marked members) is not necessarily the longest possible chain.

We address search for complex structured RNA families with the new concept of RNA secondary structure descriptors (SSD for short). SSDs use the information of multiple ordered RSSPs derived from the decomposition of an RNA's secondary structure or from the consensus secondary structure of a multiple sequence-structure alignment of related RNAs into stem-loop-like structural elements. Such consensus secondary structures for multiple RNAs can be computed with a variety of programs following one of the three strategies introduced in [41]. Namely: (A) alignment of the sequences followed by joint folding [42-45], (B) Sankoff style [8] simultaneous alignment and folding [10,12,46,47], and (C) individual folding of the sequences followed by alignment of their structures [7,48,49]. In the following we make the concept of SSDs more precise. Let *A *= *A*_1_, *A*_2_,..., *A_L _*be a sequence of non-overlapping alignment blocks. These alignment blocks are excised from a multiple sequence(-structure) alignment and represent regions of the molecule that fold into stem-loop-like structures or remain unfolded. The indexing from 1 to *L *reflects their order of occurrence in the alignment. Hence *A *represents a sequential decomposition of the molecule's secondary structure (in 5' → 3' direction) into regions, each of which can be described by an RSSP. See Figure 6(A) for an example.

An SSD  of length *L *is a sequence of *L *RSSPs  where  denotes the RSSP describing *A_i_*, *i *∈ [1, *L*]. The order ≪ of the RSSPs in  is imposed by the order of the corresponding alignment blocks. By *l_i _*and *r_i _*we denote the start and end positions of *A_i _*in the multiple alignment, respectively. In practice,  can be obtained from multiple sequence-structure alignments of related RNA sequences (i.e., of an RNA family) as they are available in databases like Rfam [3,4]. A match to  is a non-overlapping sequence of matches for some or all of the RSSPs in  in their specified order. We will now make this more precise.

Consider an RNA SSD  with total order ≪. Let  be the set of all matches for all RSSP from  in sequence *S *of length *n*. A match is represented by a pair  such that  matches at position *p *in *S*. With each  in  we associate a positive weight  which can be defined by the user. This weight allows to quantify the expressiveness of  and/or its significance. For example,  can be the length of  or it might be derived from the number of non-ambiguous nucleotides in  or the probability of obtaining a match for  just by chance assuming a certain (mono-)nucleotide background distribution. We say that matches  and  are *collinear*, written as  if  and . A *chain * for an SSD  is a sequence of matches

all from , such that  for all *i*, 1 ≤ *i *≤ *k *- 1.

There are two modes to score chains, depending on the nature of the search problem. If the multiple sequence-structure alignment our SSD is derived from and the searched sequences have comparable length, we want the chain to cover as much as possible of the sequence and we define the *global chain score *for chain  as follows:(2)

Then, the global chaining problem is to find a chain  with maximum global chain score. If we are searching in a whole genome or chromosome for a relatively short structural RNA, we are interested in local chains covering only parts of the genome or chromosome. Then we have to penalize gaps using a penalty function *g *and thus the *local chain score *is defined by(3)

where(4)

To solve the local chaining problem we use our own implementation of a fast local chaining algorithm described in [50] with modified gap costs. While the algorithm of [50] penalizes gaps by the sum of their lengths, our solution is based on the difference between their observed lengths (in the chain of matches) and their expected lengths (as given by the multiple alignment of the family), confer Equation 4. This algorithm runs in *O*(*q *log *q*) time where *q *is the size of .

To solve the global chaining problem we have developed a new efficient chaining algorithm described next.

#### An improved method for global RSSP match chaining

So far our description was based on a single sequence. However, the results described below are based on a large set of sequences *S*_1_,..., *S_k _*as it occurs when searching a large sequence database. I.e. in case of databases like Rfam *k *can be in the range of millions. To handle these, we concatenate the single sequences with separator symbols and construct the affix array for the concatenation. For a given SSD , all RSSPs , 1 ≤ *i *≤ *L*, are matched one after the other using fast bidirectional search on the affix array. This results in match sets  for RSSP . *L *is typically in the range of tens while the number of RSSP matches for a particular sequence *S_j _*is in the order of hundreds to thousands if *S_j _*is an mRNA or complete genome sequence. For each match *f *the following information is recorded:

• The ordinal number *i *of the RSSP  involved in *f*. This is denoted by *f.rssp*.

• The length of the RSSP involved in *f*. This is denoted by *f.length*.

• The number *j *of the sequence *S*_*j *_*f *occurs in. This is denoted by *f.seqnum*.

• The starting position of *f *in *S_j_*. This is denoted by *f.pos*.

• The weight  of *f*. The weight of *f *is denoted by *f.weight*.

In an initial sorting step the union  of all match sets , 1 ≤ *i *≤ *L*, is sorted in ascending order of *f.seqnum*. Matches with identical sequence numbers are sorted in ascending order of the ordinal number of the RSSP, i.e., by *f.rssp*. Suppose that b* is the size of . As there are at most b* sequences with at least one RSSP match, the sorting according to the sequence numbers can be done in  time and  space using the counting sort algorithm [51]. Here, k* is the number of sequences with at least one RSSP match. As k* ≤ b*, the sorting requires  time and space. We obtain disjoint subsets , 1 ≤ *j *≤ *k*, where  is the set of all matches in  matching a substring of *S_j_*. As  is ordered by the ordinal number of the RSSP and the counting sort algorithm is stable, the sets  are also sorted by the ordinal number of the RSSPs. Let  denote the matches  such that *f.rssp *= *i*. In a second sorting step, each  is sorted according to the starting position of the matches. As this is a typical integer sorting problem, it requires  time, where *b_j, i _*is the size of . Altogether, the two initial sorting steps can be performed in  time.

For all *S*_1_, *S*_2_,..., *S_k _*one now solves independent chaining problems for sets , 1 ≤ *j *≤ *k*, of matches sorted according to the ordinal number of the RSSP and the starting position of the matches in *S_j_*. Let *j *be fixed, but arbitrary. For each match , the weight *f.weight *is positive. Hence, an optimal chain ends with a match *f *such that there is no match *f' *satisfying *f *≪ *f'*. Similarly, an optimal chain begins with a match *f' *such that there is no match *f *satisfying *f *≪ *f'*.

The chaining problem is solved by a dynamic programming algorithm which tabulates for all matches  the maximum score *f'.score *of all chains ending with *f'*. In addition, it computes the predecessor *f'.prec *of *f' *in a chain with maximum score ending with *f'*. To obtain *f'.score*, one has to maximize over all matches *f *such that *f.rssp < f'.rssp *and *f.pos *+ *f.length *- 1 <*f'.pos*. This is a two dimensional search problem. As the matches in  are already sorted according to the first dimension (i.e., by the ordinal number of the RSSP), one can reduce it to a one dimensional sorting problem. This has already been observed in [50], and led to the development of an algorithm solving the chaining problem in , where *b *is the number of matches in . However, the algorithm of [50] was developed for chaining pairwise sequence matches. The RSSP chaining problem is a special instance of this problem: the first "sequence" consists of the positions 1,..., *L*, and a match for RSSP  is a match of length one to position *i*. Moreover, matches at position *i *in the first sequence can be treated as being of equal length because they are matches to the same RSSP . In addition to this, our initial sorting step delivers, for all *i*, 1 ≤ *i *≤ *L*, the matches in  in sorted order according to the starting position in *S_j_*. All these properties allow us to simplify and improve the algorithm of [50] in the following aspects:

• While the algorithm of [50] requires a dictionary data structure with insert, delete, predecessor, and successor operations running in logarithmic time (e.g., an AVL-tree or a red-black tree [51]), our approach only needs a linear list, which is much easier to implement and requires less space.

• While the algorithm of [50] requires an initial sorting step using  time, our method only needs  time for this step. Note that the *b_j, i _*satisfy .

• While the algorithm of [50] solves the chaining problem for  in  time, our approach runs in  time. If *L *is considered to be a constant, the running time becomes linear in *b*, where .

To explain our algorithm, let *i*, 1 ≤ *i *≤ *L *be arbitrary but fixed and assume that all match sets  have been processed. In a first loop over the sorted matches in  one determines the score of the matches. In a second loop, one inserts them into a linear list if necessary. The linear list contains a subset of the previously processed and scored matches. This split of the computation into two loops is different from the algorithm of [50] where the scoring and insertions are interweaved in one loop, requiring an extra array of length 2*b *containing references to the matches. The separation into two loops allows us to get rid of this extra array.

Now consider the first loop over all elements in  in sorted order of the match position in *S_j_*. Let *f' *be the current element. At this point, all matches *f *such that *f.rssp < f'.rssp *have been processed already. In particular, the score *f.score *and the previous match (if any) in an optimal chain ending with *f *has been determined. Among the processed matches we only have to consider those matches *f *satisfying *f.pos *+ *f.length *- 1 <*f'.pos*. If there is such a match, one takes the one with maximal score, say *f*. Then, the optimal chain ending with *f' *contains the previous match *f*, and the score is *f'.score *= *f'.weight *+ *f.score*. If there is no such match, then the optimal chain ending with *f' *only consists of *f' *and *f'.score *= *f'.weight*.

Now consider the second loop over all elements in  for which the scores and predecessor matches (if any) are already determined. Let *f' *be the current element to be inserted. As explained in the previous case, one has to make sure that, among the processed matches, one can efficiently determine the match *f *with the maximum score such that *f.pos *+ *f.length *- 1 is smaller than some value depending on *f'*. The processed matches are stored in a linear list which is sorted in ascending order of the position of the matches in *S_j_*. Let ≺*_pos _*denote this order, that is *f *≺_*pos *_*f" *if and only if *f.pos *+ *f.length < f*"*.pos *+ *f".length *for any matches *f *and *f"*. If for two processed matches *f *and *f" *one has *f.pos < f*"*.pos *and *f.score > f*"*.score*, then an optimal chain does not include *f"*. Each chain that uses *f" *can also use *f *and increase the chain score. As a consequence, one has to take care that *f" *is not inserted into the linear list or it is deleted if it was inserted earlier. In this way, *f *≺_*pos *_*f" *always implies *f.score *≤ *f".score *for two matches *f *and *f" *in the linear list. As the elements to be scored in the first loop and to be inserted in the second loop are ordered in the same way as the elements in the linear list, one can perform the scoring and the insertion loop (which also may involve deletions) by merging two lists of length *l*_1 _and *l*_2 _in  time where *l*_1 _is the number of matches to be scored and inserted and *l*_2 _is the length of the linear list involved. Let . As *l*_1 _+ *l*_2 _≤ *b*, one obtains a running time of  for each set . As there are *L *such sets, the running time is .

## Results

### Implementation and computational results

We implemented (1) the algorithms necessary for affix array construction, (2) the fast bidirectional search of RSSPs using affix arrays as sketched in Algorithm 2 (hereinafter called *BIDsearch*), (3) an online variant operating on the plain sequence (hereinafter called *ONLsearch*) for validation of *BIDsearch *and reference benchmarking, and (4) the efficient global and local chaining algorithms. Algorithm *ONLsearch *shifts a window of length *m *= |*RSSP*| along the sequence of length *n *to be searched and compares the substring inside the window with the RSSP from left to right until a mismatch occurs. Hence, it runs in  time in the worst and  time in the best case. Algorithms *BIDsearch *and *ONLsearch *were implemented in the program *afsearch*. The *afconstruct *program makes use of routines from the *libdivsufsort2 *library (see http://code.google.com/p/libdivsufsort/) for computing the suf_F _and suf_R _tables in  time. For the construction of the lcp_F _and lcp_R _tables we employ our own implementation of the linear time algorithm of [36]. Tables aflk_F _and aflk_R _are constructed in  worst-case time with fast practical construction time due to the use of the skip tables skp_F _and skp_R _[37]. The programs were compiled with the GNU C compiler (version 4.3.2, optimization option -O3) and all measurements were performed on a Quad Core Xeon E5410 CPU running at 2.33 GHz, with 64 GB main memory (using only one CPU core). To minimize the influence of disk subsystem performance the reported running times are user times averaged over 10 runs. Allowed base pairs were canonical Watson-Crick (A, U), (U, A), (C, G), (G, C), and wobble (G, U), (U, G), unless stated otherwise.

#### Affix array construction times

**Figure 7:**
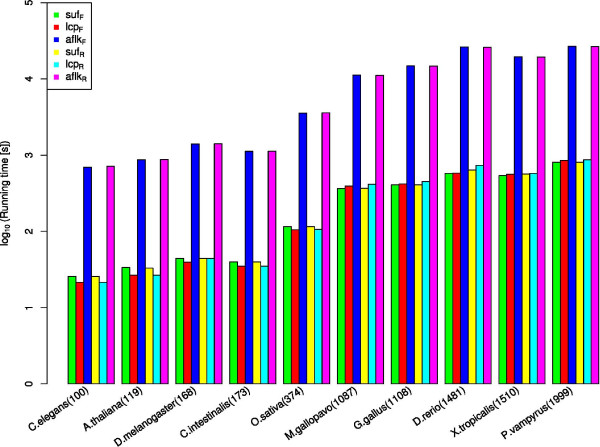
**Experiment 1: Running times for affix array construction for genomes of different model organisms**. Genome sizes are given for each organism in megabases in brackets. We measured the running time in seconds for all tables the affix array consists of (y-axis, log_10 _scale). Total construction times were in the range of ~ 25 minutes for *C. elegans *up to 15.7 hours for *P. vampyrus*.

In a first experiment we constructed the affix array for genomes of selected model organisms of different sizes and stored it on disk. We measured the total running times needed by *afconstruct *to construct each table comprising the affix array. See Figure 7 for the results of this experiment. The total size for each table is given in Additional file 1 Table S2. Construction times were in the range of 25 minutes for the *C. elegans *genome containing ~ 100 megabases to 15.7 hours for the ~ 2 gigabase genome of the megabat *P. vampyrus*.

We also measured the running time of *afconstruct *to construct the affix array for a set of 3,192,599 RNA sequences with a total length of ~ 622 MB compiled from the full alignments of all Rfam release 10.0 families. The construction and storage on disk required 126 minutes. In the following we refer to this dataset as RFAM10 for short.

#### Influence of loop length on search performance

**Figure 8:**
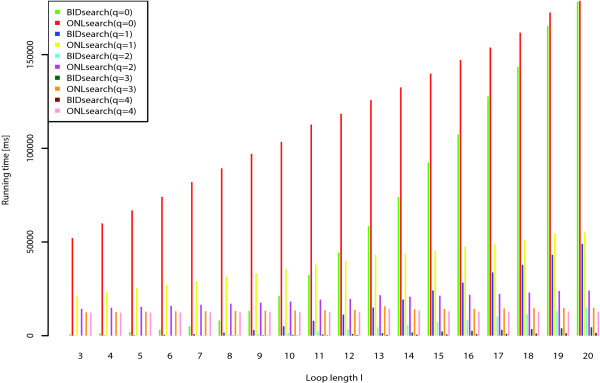
**Experiment 2: Influence of loop length and number of non-ambiguous characters in loop region on total running time of *BIDsearch *and *ONLsearch***. We measured the running time in milliseconds to search with artificial RSSPs with loops of varying length *l *∈ {3, *... *, 20} on ~ 622 MB of RNA sequence data. For each loop length *l *we also varied the number *q *∈ {0, *... *, 4} of non-ambiguous nucleotides in the loop. The used RSSPs had a fixed stem length of 7. For more details on this experiment see corresponding text.

In a second experiment we investigated the influence of the loop length and the number of non-ambiguous characters in the loop of an RSSP on the running time of *BIDsearch *and *ONLsearch*. For this experiment we constructed artificial RSSPs with a fixed stem length of 7 and a loop length *l *varying from 3 to 20. For each loop length, we also varied the number of consecutive non-ambiguous characters *q *from 0 to 4. For *q *= 0 this means that the RSSP contains structural constraints only. That is, for *q *= 0 and *l *= 5 the used RSSP matches all substrings that are able to fold into a stem-loop structure with loop length 5 and stem length 7. Such a pattern is written in dot-bracket notation as ( ( ( ( ( ( ( . . . . . ) ) ) ) ) ) ). Allowed base pairs were (A, U), (U, A), (C, G), and (G, C). We measured the time needed by *BIDsearch *and *ONLsearch *to search for these patterns in the RFAM10 dataset. Results are given in Figure 8. In this experiment *BIDsearch *performed very well and was faster than *ONLsearch *for all parameter combinations. We also investigated the influence of different stem length (data not shown here) and found that the impact on the total running time is negligible. We observe that the advantage of *BIDsearch *over *ONLsearch *decreases with increasing loop length *l *for fixed *q*. We explain this behavior with the increasing number of affix-intervals that have to be processed for finding all different substrings of the sequences that match the RSSP. However, even for an RSSP with loop length *l *= 20 containing only structural constraints (*q *= 0), *BIDsearch *is still faster than *ONLsearch*. We further notice that the number of non-ambiguous characters in the loop region has a strong influence on the running time of *BIDsearch*. That is, by specifying only a few conserved nucleotides in the RSSP's loop region, the running time of *BIDsearch *is reduced dramatically. For an example of this effect, see the running times of *BIDsearch *in Figure 8 for parameters *l *= 15 and *q *∈ {2, 3, 4}. This renders *BIDsearch *in particular useful for searching with RSSPs with moderate loop length or existing sequence conservation in the loop region. The speedup factors measured in this experiment were in the range from 1.001 to 78.1 for *q *= 0 and from 9.28 to 11 × 10^3 ^for *q *= 4. Table 1 gives more details on the speedups of *BIDsearch *over *ONLsearch *for all investigated combinations of *q *and *l*.

**Table 1:** Experiment 2: Obtained speedup of *BIDsearch *over *ONLsearch *for different loop length *l *∈ {3,..., 20} and number of non-ambiguous characters in the loop region *q *∈ {0, *... *, 4}

*l*	3	4	5	6	7	8	9	10	11
*q *= 0	78.10	48.64	35.42	23.55	16.35	11.01	7.31	4.89	3.48
*q *= 1	329.81	180.45	105.67	57.41	33.75	19.20	11.30	7.14	4.81
*q *= 2	749.94	418.65	227.45	121.80	67.81	36.99	21.44	12.73	8.41
*q *= 3	2,345.17	1,169.53	653.31	353.49	188.34	103.34	56.59	33.08	20.79
*q *= 4	11,045.75	3,638.14	2,144.8	1,132.53	610.63	338.77	184.56	106.11	64.93

***l***	**12**	**13**	**14**	**15**	**16**	**17**	**18**	**19**	**20**

*q *= 0	2.67	2.15	1.79	1.51	1.37	1.20	1.13	1.07	1.00
*q *= 1	3.58	3.13	2.28	1.89	1.68	1.46	1.35	1.27	1.12
*q *= 2	5.96	4.88	3.64	2.94	2.57	2.19	2.02	1.82	1.63
*q *= 3	14.27	11.88	8.25	6.50	5.53	4.74	4.19	3.76	3.34
*q *= 4	43.09	35.23	25.74	19.52	15.91	13.25	11.75	10.32	9.28

For the parameter combination *l *= 3, *q *= 4 also one character of the stem was specified.

#### Searching large sequence databases

To measure the performance of *BIDsearch *for non-artificial real-world RSSPs, we manually compiled a set of 397 RSSPs describing 42 highly structured RNA families taken from the RFAM10 database. These were all families with a consensus secondary structure containing at least 5 stem-loop substructures. We measured the running time needed by *BIDsearch*, *ONLsearch*, and the widely used tools *RNAMotif *[13] and *RNABOB *[15] to search for these 397 RSSPs in the RFAM10 dataset. As expected, all tools delivered identical results. However, while it took *BIDsearch *less than 50 seconds to search for the 397 patterns as shown in Table 2, *RNABOB *and *RNAMotif *needed more than 2.5 and 3.2 hours respectively to complete the same task. This made for a speedup factor of 196.5 (254.7) for *BIDsearch *over *RNABOB *(*RNAMotif*). Even if we include the time needed for affix array construction, *BIDsearch *is still faster than *RNABOB *and *RNAMotif*.

**Table 2:** Experiment 3 (A): Running times in seconds needed by the programs to search for 397 RSSPs describing 42 RFAM10 families in ~ 622 megabases of RNA sequence data.

*BIDsearch*	*ONLsearch*	*RNAMotif*	*RNABOB*
46.1(1)	6,203(134.5)	11,745(254.7)	9,061(196.5)

For each program the speedup factor of *BIDsearch *over the particular program is given in brackets.

We also investigated the distribution of speedup factors obtained by *BIDsearch *when searching for the 397 RSSPs. We observed that *BIDsearch *is more than 50,000 times faster than *RNABOB *and *RNAMotif *for the majority of the patterns and that the total search time required by *BIDsearch *is dominated by only a small number of patterns. These patterns describe large unconserved loop regions. See Figure S3 in Additional file 1 for a graphical visualization of the distribution of speedup factors.

#### Scaling behavior of bidirectional pattern search using affix arrays

**Figure 9:**
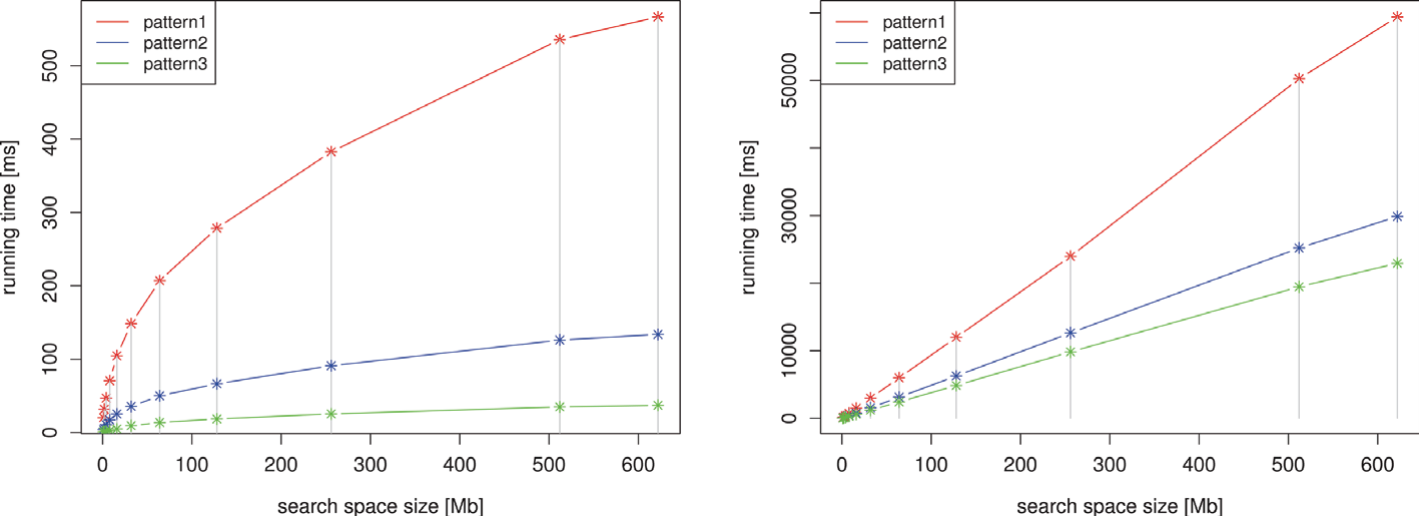
**Scaling behavior *BIDsearch *(left) and *ONLsearch *(right)**. We measured the running time needed to search with three different patterns on random subsets of RFAM10 of different sizes. For details, see main text.

In a further experiment we investigated the scaling behavior of *BIDsearch *and *ONLsearch *for an increasing size of sequences to be searched. For this, we searched with different RSSPs on random subsets of RFAM10 of different sizes and measured the running time for both algorithms. The results are given in Figure 9. Here pattern1 is an RSSP containing only structural constraints. It describes a stem-loop with loop length 4, stem length 10 and no specified nucleotides in the loop region. The RSSP pattern2 (pattern3) only differ from pattern1 by containing one (two consecutively) non-ambiguous nucleotides in the loop region.

In this experiment *BIDsearch *clearly showed a sublinear scaling behavior, whereas *ONLsearch *scaled only linearly. It took *BIDsearch *only 566.8 (pattern1), 133.8 (pattern2), and 37.1 (pattern3) milliseconds to search the whole RFAM10 dataset. The obtained speedups of *BIDsearch *over *ONLsearch *were in the range from 4.63 (*1 MB subset*) to 104.79 (*full *RFAM10) for pattern1, from 12.23 (*1 MB subset*) to 223.18 (*full *RFAM10) for pattern2, and from 35.0 (*1 MB subset*) to 618.37 (*full *RFAM10) for pattern3. We observe again that the specification of only one or two nucleotides in an RSSP's loop region considerably reduces the running time of the *BIDsearch *algorithm.

#### RNA family classification by global chaining of RSSP matches

**Figure 10:**
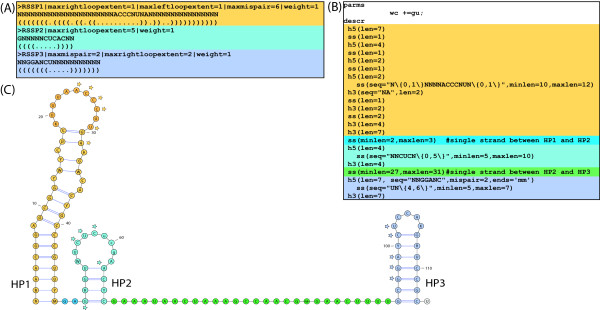
**Descriptors for the OxyS RNA family.** (A) Secondary structure descriptor for the family of OxyS RNAs in *Structator *syntax. The SSD consists of RSSPs RSSP1, RSSP2, and RSSP3 describing the three stem-loop structures (HP1, HP2, and HP3, see (C)) of this small non-coding RNA. (B) *RNAMotif *descriptor for the same structural elements. (C) Consensus secondary structure of the OxyS RNA family as drawn by *VARNA *[55]. Sequence information (non-wildcard nucleotides) used in both descriptors are marked with an asterisk. Observe that both descriptors use predominantly structure and very little sequence information.

To demonstrate the effect of global chaining of RSSP matches, we searched with an SSD built for the Rfam family of OxyS RNAs (Acc.: RF00035). OxyS is a small 109-nucleotide long non-coding RNA which is included in response to oxidative stress in *E. coli *[52]. Members of this family fold into a characteristic secondary structure consisting of three stem-loop substructures, referred to as HP1, HP2, and HP3 in Figure 10(C). From the three stem-loops we derived three descriptors called RSSP1, RSSP2, and RSSP3, which constitute the SSD describing this family. We note that in this experiment the RSSPs were constructed to guarantee high specificity and thus to minimize the number of false positives. For the SSD specified in *Structator *syntax, see Figure 10(A). Searching for this SSD in RFAM10, *Structator *delivers 8,619 matches for RSSP1, 1,699 matches for RSSP2, and 142,219 matches for RSSP3. Instead of reporting these matches, *Structator *computes high-scoring global chains for each sequence containing matches to all three RSSPs. The chains and the sequences they occur in are reported in descending order of the chain score. This procedure resulted in 61 sequences, all belonging to the OxyS family which contains 115 members in total. Hence, by considering only high-scoring chains all the spurious RSSP matches were eliminated. We also described the same three stem-loops in a format compatible with *RNAMotif *(see Figure 10(B)). A search on RFAM10 with this descriptor returned exactly the same 61 sequences. However, *Structator *operating in *BIDsearch *(*ONLsearch*) mode with subsequent global chaining of RSSP matches needed only 3.9 (122.5) seconds to identify all family members, whereas *RNAMotif *needed 84.7 seconds. The search times for *Structator *include 0.05 seconds required for the chaining.

We also employed global chaining to detect members of the structurally more complex family of Citrus tristeza virus replication signal (Rfam Acc.: RF00193). Therefore we built an SSD comprising 8 RSSPs, describing 8 of 10 stem-loops the molecule is predicted to fold into. For more information on the molecule's secondary structure and the used descriptor, see Additional file 1 Figure S4. Using *Structator *operating in *BIDsearch *(*ONLsearch*) mode and global chaining of RSSP matches it took only 1.3 (138.7) seconds to search RFAM10 with this SSD, where 0.06 seconds were required for the chaining. The computed global chains with a minimum length of 5, computed from the 184,199 single RSSP matches, were ranked according to their global chain score. We observe that the sequences containing the 37 highest scoring chains are exactly all 37 members of the family.

In addition we measured the performance of *Structator *using global chaining for RNA family classification with manually compiled SSDs for 42 Rfam families. For the results of this experiment see Additional file 1 Table S4.

#### Searching whole genomes using local chains of RSSP matches

**Figure 11:**
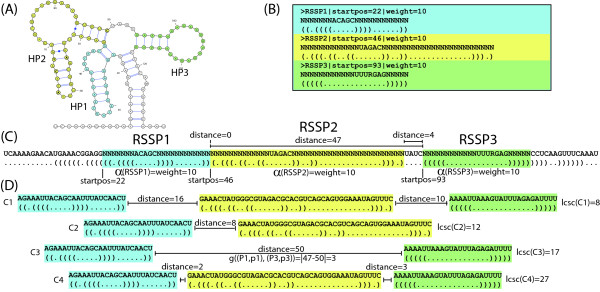
**An example of local chains of RSSP matches**. (A) Consensus secondary structure visualized with the *VARNA *program of the HAR1F RNA family showing stem-loops HP1, HP2, and HP3. (B) SSD consisting of RSSP1, RSSP2, and RSSP3 in *Structator *syntax describing the three stem-loop regions of HAR1F. (C) Regions of HAR1F described by the RSSPs, including distances *l*_*i*+1 _- *r*_*i*_, 1 ≤ *i *< 3, between neighbored RSSPs and RSSP weights *α*(RSSP*_i_*), 1 ≤ *i *≤ 3. (D) Examples of local chains , 1 ≤ *i *≤ 4 found with the SSD, showing, in each chain, the distance between RSSP matches and their local chain score . Gap cost computation according to Equation (4) is shown exemplary for the two RSSP matches of chain .

As an example of searching a complete genome or whole chromosomes for non-coding RNAs, we searched for the RNA gene Human accelerated region 1F (HAR1F) on both strands of the human genome sequence. HAR1F is one of 49 regions in the human genome that differ significantly from highly conserved regions of the chimpanzee [53]. The consensus structure of the HAR1F family in Rfam (Acc.: RF00635) contains three stem-loop regions, denoted HP1, HP2, and HP3 in Figure 11(A). From these regions, we built an SSD for the family with RSSPs RSSP1, RSSP2, and RSSP3, shown in Figure 11(B). Since we were searching on complete chromosomes, we only wanted to consider RSSP matches that occurred at a similar distance to each other w.r.t. to the distances of the corresponding descriptors in the SSD. Therefore, unlike in the previous experiment where we searched for global chains of RSSP matches, we now computed high-scoring local chains. Gap costs were computed according to Equation (4) and we used an RSSP weight *α*(RSSP*_i_*) = 10, for 1 ≤ *i *≤ 3. Affix array construction for all human chromosomes was accomplished in 12.6 hours by *afconstruct*. We searched with *Structator *for the three RSSPs and found 15,090, 1,578, and 14,491 matches for RSSP1, RSSP2, and RSSP3, respectively. For these RSSP matches we computed local high-scoring chains (see Figure 11(D)). Chains  were ranked according to their local chain score . We observed that the highest-scoring chain corresponds to the correct location of the gene on chromosome 20. Using *BIDsearch *(*ONLsearch*) this task needed 3.1 (633.4) seconds only, including 0.02 seconds for the chaining. *RNAMotif *also found a single match corresponding to the correct location of the gene, but needed 274.7 seconds. See Figure S5 in Additional file 1 for the used *RNAMotif *descriptor.

#### Comparison of implementations of bidirectional pattern search

In the last experiments we compared *Structator'*s running time using using *BIDsearch *with the time needed by a recently published bidirectional pattern search implementation for the same task. The implementation of [54], to which we refer as *BWI*, uses a compressed data structure called bidirectional wavelet index. We remark that *BWI *can only search with a small set of hard-coded patterns, i.e., the user cannot use it to search with his/her own patterns. Moreover, unlike *Structator*, which provides a full command line interface with many configurable options (see section about the software package), *BWI *reports neither matching substrings nor matching positions (which is known to be the most time consuming part when querying compressed index structures [26]). It only outputs the search time of individual patterns and the number of matches. Thus, it serves rather as a prototype implementation of the concepts introduced in [54]. Nevertheless, since it also makes use of bidirectional search, we compared *BWI *with *Structator *using *BWI'*s hard-coded patterns. See Table 3 for the results. Details of the database and patterns are as previously described [54]. We noticed that *BIDsearch *was faster than *BWI *for matching all patterns by up to factor 2, hence making it preferable when speed is most important. However, we note that *BWI'*s compressed wavelet index consumes significantly less memory than *Structator'*s affix array index, which would make *BWI *preferable in cases where space consumption is critical. See Table S3 in Additional file 1 for the memory required by *BWI'*s index for different genomes. We also measured the speedup of *Structator *running in *BIDsearch *mode over *ONLsearch *and compared the results with previously reported measurements [27]. Because the implementation used there is not available (personal communication with the author), we calculated relative speedups based on the reported absolute running times. Details on this experiment are given in Additional file 1 Section S2.

**Table 3:** Search time comparison between *Structator'*s *BIDsearch *and an implementation, here called *BWI*, of bidirectional search using the wavelet tree data structure described in [54].

	hairpin1	hairpin2	hairpin4	hloop(5)	acloop(5)	acloop(10)
*BWI*	10,484	64	612	26,413	896	420
*BIDsearch*	8,325	32	330	16,768	511	295
*BIDsearch *vs. *BWI*	1.26	2	1.85	1.58	1.75	1.42

Search times are in milliseconds. The last row shows the speedup of *BIDsearch *over *BWI*.

### Structator software package

*Structator *is an open-source software package for fast database search with RNA structural patterns implementing the algorithms and ideas presented in this work. It consists of the command line programs *afconstruct *and *afsearch*.

*afconstruct *implements all algorithms necessary for affix array construction, namely a lightweight suffix sorting algorithm for construction of the suffix arrays suf_F _and suf_R_, the algorithm for construction of tables lcp_F _and lcp_R _[36], and the algorithm for computation of the affix link tables aflk_F _and aflk_R_. The program constructs all or if necessary only some of the tables of the affix array for a target database provided in FASTA format and stores them on disk. Therefore the program can also be used to compute only the tables needed for a traditional enhanced suffix array [34]. *afconstruct *can handle RNA as well as DNA sequences. Moreover, it supports the transformation of input sequences according to user-defined (reduced) alphabets and allows the index construction for transformed sequences. Such personalized alphabets are easily specified in a text file.

**Figure 12:**
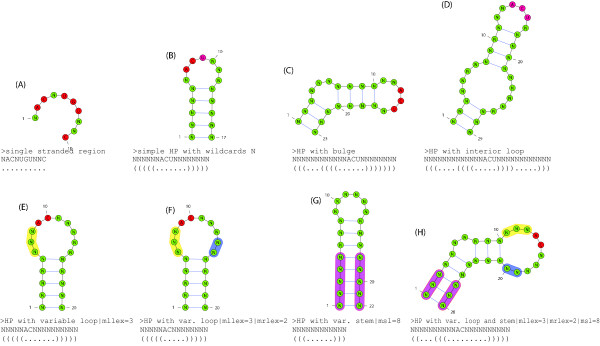
**Supported structural patterns and corresponding pattern definitions in *Structator *syntax**. Non-ambiguous nucleotides are marked in red. Positions containing ambiguous nucleotides, denoted here with character N, are marked in green and can contain any nucleotide from . Maximal allowed left and right extensions of the loop region of a pattern as specified by parameters *maxleftloopextent (mllex) *and *maxrightloopextent (mrlex) *are marked in yellow and blue, respectively. Allowed possible extensions of a pattern's stem region as specified by parameter *maxstemlength (msl) *are marked in purple. As an example for the semantics of the parameter *msl *consider pattern (G): it matches all substrings of the searched sequence that are able to fold into a stem-loop structure with loop length 6 and stem length between 3 and 8. For further details see corresponding text.

*afsearch *is the program for performing structural pattern matching. That is, it searches (ribo)nucleic acid sequence databases for entries that can adopt a particular secondary structure. For an overview of the supported RNA sequence-structure patterns (RSSPs), see Figure 12. The simplest RSSP describes a single-stranded region, where ambiguous (not well-conserved) nucleotides can be specified with IUPAC characters. All ambiguous IUPAC characters are hard-coded in *afsearch*, e.g. N standing for nucleotides A, C, G, and U (and T) and R standing for A and G. Besides fixed-length RSSPs with or without ambiguous characters (Figure 12(A) until 12(D)), also RSSPs describing loop or stem regions of variable size (Figure 12(E) until 12(H)) are supported. More precisely, one can specify with parameters *maxleftloopextent (mllex) *and *maxrightloopextent (mrlex) *a variable number of allowed extensions to the left (nucleotides marked in yellow in Figure 12(E)) and/or to the right (nucleotides marked in blue in Figure 12(F)) for the specified loop pattern. Variable stem sizes can be addressed with parameter *maxstemlength (msl) *(see regions marked in pink in Figure 12(G)). Also supported is the combination of variable loop and stem size (see Figure 12(H)) and a maximal number of allowed mispairings in the stem region. All these different RSSPs can be specified by the user in a text file which use, as shown in Figure 12, an expressive but easy to understand pattern syntax. For additional details on the supported patterns see the corresponding section in the *Structator *user manual. *afsearch *also permits user-defined base pairing rules. That is, the user can define an arbitrary subset from  as valid pairings. This ensures a maximum of flexibility. For example, the standard canonical Watson-Crick pairings as well as non-standard pairings such as G-U can be specified.

The search is performed efficiently on a pre-computed affix array. *afsearch *implements the bidirectional index-based search algorithms *BIDsearch *and the online algorithm *ONLsearch *operating on the plain sequence, both extended to support patterns with variable loop size and/or stem length. Further, it implements the methods for fast global and local chaining of RSSP matches. The search with RSSPs can be performed on the forward and, in case of nucleotide sequences, also on the reverse strand. Searching on the reverse strand is implemented by reversal of the RSSP and transformation according to Watson-Crick base pairing. Hence it is sufficient to build the affix array for one strand only.

RSSP matches can be reported directly by *afsearch *or can be used as input for the computation of high-scoring global or local chains of matches. Computed chains resemble the order of the RSSPs given in the pattern file and are reported in descending order of their chain score. This allows the description of complex secondary structures with our new concept of secondary structure descriptors (SSDs). This is done by simply specifying a series of RSSPs in the pattern file describing the stem-loop substructures the RNA molecule is composed of in the order of their occurrence in 5' to 3' direction. To incorporate different levels of importance or significance of an RSSP into SSD models and subsequently in the computation of chain scores, RSSP specific weights can be defined in the pattern file. This is particularly useful in the context of RNA family classification where the used SSD may be derived from a multiple sequence-structure alignment or a consensus structure-annotated multiple sequence alignment. Here, it permits the assignment of higher weights to RSSPs describing highly conserved functionally important structural elements occurring in a family of RNAs, and lower weights to RSSPs describing less conserved substructures that occur only in certain members of the family.

The output format of *afsearch *contains all available information of a match or chain of matches, either in a human-readable, or a tab-delimited format. Moreover, *afsearch *can also report matches in BED format. This allows a direct visualization of the results in e.g. the UCSC genome browser.

## Discussion and conclusion

We have presented a method for fast index-based search of RNA sequence-structure patterns (RSSPs), implemented in the *Structator *software. As part of the software, we give the first publicly available implementation of bidirectional pattern search using the affix array data structure. For the majority of biologically relevant RSSPs, our implementation of *BIDsearch *shows superior performance over previous programs. In a benchmark experiment on the Rfam database, *BIDsearch *was faster than *RNAMotif *and *RNABOB *by up to two orders of magnitude. Furthermore, in a comparison between *BIDsearch *and the program of [54], which works on compressed index data structures, *BIDsearch *was faster by up to 2 times. We observed that for RSSPs with long unconserved loop regions, the advantage of *BIDsearch *over *ONLsearch *decreases. For such cases, *Structator *can also employ *ONLsearch *on the plain sequence data. As a further contribution, we presented for the first time a detailed complexity analysis of bidirectional search using affix arrays. While bidirectional search does not does not improve the worst-case time complexity compared to online search, in practice it runs much faster than online search algorithms and the running time scales sublinearly with the length *n *of the searched sequences.

Our implementation of the affix array data structure requires only 18*n *bytes of space. This is a significant space reduction compared to the ~ 45*n *bytes needed for the affix tree. With the program *afconstruct *we present for the first time a command line tool for the efficient construction and persistent storage of affix arrays that can also be used as a stand-alone program for index construction.

With the new concept of RNA secondary structure descriptors (SSDs) combined with fast global and local chaining algorithms, all integrated into *Structator*, we also introduce a powerful technique to describe RNAs with complex secondary structures. This even allows to effectively describe RNA families containing branching substructures like multi-loops, by decomposition into sequences of non-branching substructures that can be described with RSSPs. Compared to programs like *RNAMotif *, *Structator'*s pattern description language for RSSP formulation is simple but powerful, in particular in combination with the SSD concept. Beyond the algorithmic contributions, we provide with the *Structator *software distribution a robust, well-documented, and easy-to-use software package implementing the ideas and algorithms presented in this manuscript.

## Availability

The *Structator *software package including documentation is available in binary format for different operating systems and architectures and as source code under the GNU General Public License Version 3. See http://www.zbh.uni-hamburg.de/Structator for details.

## Authors' contributions

FM implemented the presented algorithms and wrote parts of the manuscript and the *Structator *manual. SK developed and implemented the RSSP chaining algorithms and contributed to the manuscript. SW provided supervision and wrote parts of the manuscript. MB initiated the project, provided supervision and guidance, designed/performed the experiments and wrote large parts of the manuscript. RB contributed to the introduction. All authors read and approved the final manuscript.

## Supplementary Material

Additional file 1 **Supplemental material**. Additional file 1 contains additional examples, algorithms, experiments, figures, and tables. Click here for file
