# Supplementary material for: Structator: fast index-based search for RNA sequence-structure patterns
Source: BMC Bioinformatics. 2011 May 27;12:214. doi: 10.1186/1471-2105-12-214 (PMC3154205; doi:10.1186/1471-2105-12-214)
Supplement: Additional file 1 — Supplemental material. Additional file 1 contains additional examples, algorithms, experiments, figures, and tables. [file 1471-2105-12-214-S1.PDF]

Supplemental material for the paper: Structator: fast index-based search for RNA sequence-structure patterns

Fernando Meyer Stefan Kurtz Rolf Backofen
Sebastian Will Michael Beckstette

1 An example of bidirectional RSSP search

As an example of bidirectional search for RSSPs using affix arrays, we search for the RSSP $\mathcal{Q}$ in the sequence S of Figures 2 and 3, respectively, of the main document. We recall that $\mathcal{Q} = (P, R)$ with $P =$ NNNUGCUNNN and $R =$ `(((....)))` represents a stem-loop structure of length $m = 10$ and $S =$ AUAGCUGCUGCUGCA has length 15. We start matching P in S by calling procedure *bidir-search* of Algorithm 2 as *bidir-search*$(\langle 0, 0 - [0..15], \mathsf{F}\rangle, 2, 3)$. That is, the algorithm matches the first position $P[3] =$ U of the loop region in left-to-right direction. Given that $\mathsf{X} = \mathsf{F}$ and $i < j$ (i.e. $0 < 15$) hold, it locates interval $v_x = \langle 0, 1 - [11..14], \mathsf{F}\rangle$ with $\overrightarrow{v_x} =$ U via binary search in the interval $0 - [0..15]$ of suf_F. Analogously, the following recursive calls of *bidir-search* perform right c-extensions of $u =$ U $= P[3..3]$ with characters $P[4] =$ G, $P[5] =$ C, and $P[6] =$ U, by searching in the intervals $1 - [11..14]$, $2 - [12..14]$, and $3 - [12..14]$, respectively. After these extensions, the algorithm has located the affix-interval $v_x = \langle 0, 4 - [13..14], \mathsf{F}\rangle$ representing all occurrences of $\overrightarrow{v_x} =$ UGCU in S such that $\overrightarrow{v_x}$ matches $u = P[3..6]$. We set $v = v_x$. Next, the algorithm performs a right c-extension of u with the pairing position $c \in \varphi(P[7] = \text{N})$. Therefore, it enumerates all possible v_x such that $\overrightarrow{v_x} = \overrightarrow{v}d$ for some $d \in \varphi(c)$. We observe that $v_x = \langle 0, 5 - [13..14], \mathsf{F}\rangle$ with $\overrightarrow{v_x} =$ UGCUG is the only interval satisfying these conditions and conclude $d =$ G. As an additional structural constraint, matches to positions 2 and 7 of P shall form a base pair. To fulfill this constraint the algorithm first switches the search direction by locating the reverse interval v' of v_x. The left boundary of v' is determined with a lookup in table aflk_F as $\mathsf{aflk}_\mathsf{F}[\mathsf{home}_\mathsf{F}([13..14])] = 5$ and the right boundary as 6. Further, we set $\ell_{\text{lcp}} = min\{\mathsf{lcp}_\mathsf{F}[r] \mid 13 < r \leq 14\} = 6$ and calculate the context of v' as $6 - 5 = 1$. Hence, the reverse interval of v_x is determined as $v' = \langle 1, 6 - [5..6], \mathsf{R}\rangle$ with $\overrightarrow{v}' =$ UGCUG and we set $v = v'$. Now the only interval satisfying (1) $\overrightarrow{v_x} = e\overrightarrow{v}, e \in \varphi(P[2])$, and (2) the complementarity condition between positions 2 and 7 of P, as required by the structure string R, is the interval $v_x = \langle 1, 7 - [5..6], \mathsf{R}\rangle$ with $\overrightarrow{v_x} =$ CUGCUG

representing occurrences of substrings matching $P[2..7]$. Observe that $\overrightarrow{v_x}[0] = \mathrm{C}$ and $\overrightarrow{v_x}[5] = \mathrm{G}$ can form a base pair as demanded by $R[2]$ and $R[7]$. Consequently, $\overrightarrow{v_x}$ matches $(P[2..7], R[2..7])$ and therefore we set $v = v_x$. In the next step the algorithm performs another left c-extension of $\overrightarrow{v}$ by some $c \in \varphi(P[1] = \mathrm{N})$ leading to interval $v_x = \langle 1, 8 - [5..6], \mathsf{R}\rangle$ with $\overrightarrow{v_x} = \mathrm{GCUGCUG}$ representing occurrences of substrings matching $P[1..7]$. We set $v = v_x$. To match a character $d \in \varphi(c)$ that is complementary to $\overrightarrow{v}[0] = \mathrm{G}$ the algorithm performs a right c-extension of $\overrightarrow{v}$ using a character $c \in \varphi(P[8])$. Because the context of v is larger than zero, it consumes the context and remains in table suf_R. That is, $\mathsf{X} = \mathsf{R}$. The resulting interval after performing the right c-extension is $v_x = \langle 0, 8 - [5..6], \mathsf{R}\rangle$ with $\overrightarrow{v_x} = \mathrm{GCUGCUGC}$. Observe that $\overrightarrow{v_x}[0] = \mathrm{G}$ and $\overrightarrow{v_x}[7] = \mathrm{C}$ can form a base pair and thus v_x represents occurrences of substrings of S matching $(P[1..8], R[1..8])$. We set $v = v_x$. The next operation is a left c-extension by some $c \in \varphi(P[0] = \mathrm{N})$. Hence, the algorithm enumerates all intervals v_x such that $\overrightarrow{v_x} = \overrightarrow{v}d, d \in \varphi(c)$. There are two intervals satisfying these conditions. Namely, $v_{x1} = \langle 0, 9 - [5..5], \mathsf{R}\rangle$ with $\overrightarrow{v_{x1}} = \mathrm{AGCUGCUGC}$ and $v_{x2} = \langle 0, 9 - [6..6], \mathsf{R}\rangle$ with $\overrightarrow{v_{x2}} = \mathrm{UGCUGCUGC}$. We set $v_1 = v_{x1}$ and $v_2 = v_{x2}$ and continue by processing v_1, which represents occurrences of $\overrightarrow{v_1} = \mathrm{AGCUGCUGC}$ in S. Because $\overrightarrow{v_1}$ is a unique substring of S, for the following right c-extension by some $c \in \varphi(P[9] = \mathrm{N})$ we can directly evaluate $S^\mathrm{R}[\mathsf{suf}_\mathsf{R}[5] - 1] = \mathrm{U}$. Bases $(\overrightarrow{v_1}[0] = A, U)$ are complementary, hence we set $v_x = \langle -1, 9 - [5..5], \mathsf{R}\rangle$ and observe that occurrences of substring $\overrightarrow{v_x} = \mathrm{AGCUGCUGCU}$ of S match $(P[0..9], R[0..9])$ and that the boundaries of $\mathcal{Q}$ have been reached. With this, in the following recursion the algorithm reports a matching position of $\mathcal{Q}$ via a lookup in table suf_R as $\mathsf{suf}_\mathsf{R}[5] + (-1) = 4 - 1 = 3$, where -1 is the context of v_x that has to be added to $\mathsf{suf}_\mathsf{R}[5]$. Note that, because $\mathsf{X} = \mathsf{R}$, 3 is a position in S^R. Now the algorithm backtracks to interval $\langle 0, 8 - [5..6], \mathsf{R}\rangle$ and continues to perform a right c-extension of interval v_2 by some $c \in \varphi(P[9])$. Again, $\overrightarrow{v_2} = \mathrm{UGCUGCUGC}$ is a unique substring of S and we can directly evaluate $S^\mathrm{R}[\mathsf{suf}_\mathsf{R}[6] - 1] = \mathrm{A}$. Since bases $(\overrightarrow{v_2}[0] = U, A)$ can pair, we set $v_x = \langle -1, 9 - [6..6], \mathsf{R}\rangle$ with $\overrightarrow{v_x} = \mathrm{UGCUGCUGCA}$ representing occurrences of substrings of S matching $(P[0..9], R[0..9])$. The boundaries of $\mathcal{Q}$ have been reached again and in the following recursion the algorithm reports another matching position of $\mathcal{Q}$, precisely $\mathsf{suf}_\mathsf{R}[6] + (-1) = 1 - 1 = 0$. There are no further intervals to process and the search ends. In summary, *bidir-search* has found two occurrences of $\mathcal{Q}$ in S.

2 Comparison of two implementations of bidirectional pattern search

We measured the speedup of *Structator* running in *BIDsearch* mode over *ONLsearch* and compared the results with previously reported measurements [1]. Because the implementation used by Strothmann [1] is not available (personal communication), we calculated relative speedups based on the absolute running times reported in [1]. We note that the measurements of [1] were performed on different hardware. This can, according to our experiments, significantly influence the performance of *BIDsearch*.

See Table S1 for the results of the comparison of *BIDsearch* with the method of [1]. For a description of the used RSSPs see [1]. The search was performed in the genomes of *P. horikoshii* (GenBank Acc.: NC_000961, 1.7 MB) and *E. coli* (GenBank Acc.: AC_000091, 4.5 MB), which were also used in [1]. Additionally we included with *P. vampyrus* (GenBank Acc.: ABRP00000000, 1.9 GB) a larger eukaryotic genome in this experiment.

Surprisingly, with the RSSPs ACloop(5), ACloop(10), and ACloop(15) taken from [1], which describe a loop consisting of 5 (10 and 15) repetitions of AC the speedup of the affix array based method of [1] decreased with increasing loop length. This is a behavior which is opposite to our observations (see Figure 8 of the main document). We also noticed that *BIDsearch* obtained a higher speedup when searching for RSSP Hpin2 in *E. coli* than the method of [1] but not when searching in the smaller genome of *P. horikoshii*. This observation remains unclear and cannot be further investigated due to unavailability of the implementation used in [1].

3 A bidirectional search algorithm supporting variable-length RSSPs

Algorithm 2 of the main document matches fixed-length RSSPs. We here present an extension of it also capable of matching RSSPs with loop region allowing a variable number of additional extensions with ambiguous characters N to the left and to the right. In combination, also stem region of variable length is supported. We observe that this extended version is as efficient as the original algorithm supporting fixed-length RSSPs. Additional computation time is only required for the traversal of additional affix-intervals due to the increased sensitivity.

Before describing the algorithm, we define this extension of RSSPs. A *variable-length RSSP* $\mathcal{Q}$ consists of an RSSP (P, R) and parameters *maxleftloopextent (mllex)*, *maxrightloopextent (mrlex)*, and *maxstemlength (msl)*. *mllex* and *mrlex* denote the maximum number of respective left and right extensions of the loop region specified in R and *msl* denotes the maximum number of base pairs in the stem. The minimum length of occurrences of $\mathcal{Q}$ is $m = |P| = |R|$. For examples of variable-length RSSPs, see Figure 12 (E) until (H) of the main document.

To keep the code simple, we split the original algorithm into two procedures. (i) First the loop region of a given variable-length RSSP $\mathcal{Q}$ is matched with procedure *bidir-search-loop* (see Algorithm 3, Figure S1). (ii) Next, the stem region is matched with procedure *bidir-search-stem* (see Algorithm 4, Figure S2). Note that *bidir-search-stem* is very similar to Algorithm 2 of the main document. Prior to the search for $\mathcal{Q}$, the following variables are set: *loopstart*, *minloopstart*, *loopend*, *maxloopend*, *minbps*, and *maxbps*. These variables store the following information. *loopstart* (*loopend*) stores the position of the base occurring in the left-most (right-most) position of the loop described by the structure string R in 5' to 3' direction, $minloopstart = loopstart - mllex$, $maxloopend = loopend + mrlex$, and *minbps* ($maxbps = msl$) is the minimum (maximum) number of base pairs occurring in $\mathcal{Q}$. It holds: $minloopstart \leq$

	P. horikoshii (1.7 MB)				*E. coli K12* (4.5 MB)				*P. vampyrus* (1.9 GB)			
RSSP	*ONL*	*BID*	Bvs.O	*STR*	*ONL*	*BID*	Bvs.O	*STR*	*ONL*	*BID*	Bvs.O	*STR*
Hpin1	169.61	65.59	2.59	10.26	432.94	141.84	3.05	12.17	172,913.36	9,520.39	18.16	-
Hpin2	33.34	0.27	123.48	155	88.61	0.45	196.91	99.25	34,702.63	48.85	710.39	-
Hloop(5)	214.8	166.94	1.29	14.6	552.67	372.57	1.48	18.09	219,547.76	23,958.41	9.16	-
Hloop(10)	331.96	1,412.64	0.23	2.13	842.32	3,235.11	0.26	2.43	335,928.97	248,711.65	1.35	-
ACloop(5)	59.07	4.43	13.33	182	152.87	9.91	15.43	815	64,053.16	825.79	77.57	-
ACloop(10)	58.71	1.37	42.85	4	152.12	3.45	44.09	7.24	64,136.82	391.56	163.8	-
ACloop(15)	58.67	0.89	65.92	1.3	152.01	1.86	81.73	1.38	64,199.98	278.76	230.31	-

Table S1: Comparison of speedup of *Structator*'s *BIDsearch* over *ONLsearch* (column Bvs.O) and the speedup of affix array based search over searching on the plain text as reported in [1] (column *STR*). The respective search times of *BIDsearch* (column *BID*) and *ONLsearch* (column *ONL*) are shown in milliseconds. For *P. vampyrus* only measurements for *Structator* are available.

$loopstart \leq loopend \leq maxloopend$. Note that *minloopstart* can be negative. As an example, let R = `(((....)))`, $mllex = 4$, and $mrlex = 1$. Then $loopstart = 3$, $minloopstart = -1$, $loopend = 6$, $maxloopend = 7$, and $minbps = 3$. To match $\mathcal{Q}$, procedure *bidir-search-loop* is initially called as *bidir-search-loop*$(\langle 0, 0 - [0..n], \mathsf{F}\rangle, r_0 - 1, r_0, \text{true})$, where $\langle 0, 0 - [0..n], \mathsf{F}\rangle$ is an affix-interval, r_0 is any position in the loop region of $\mathcal{Q}$, and parameter true states that the pattern can be extended to the right. Procedure *bidir-search-loop* makes a call to *bidir-search-stem* whenever substrings of minimum length $loopend - loopstart + 1$ matching the loop in the searched database are found. If $\mathcal{Q}$ has no base pairs, i.e. $msl = 0$, it instead immediately reports the matching positions. The call to *bidir-search-stem* is made as *bidir-search-stem*$(v', loopstart - 1, loopend + 1, 0)$, where v' is the affix-interval representing all occurrences of substring $\overrightarrow{v}'$ in the searched database matching the loop region of $\mathcal{Q}$, positions $loopstart - 1$ and $loopend + 1$ denote the inner-most base pair $(loopstart - 1, loopend + 1)$ of the pattern, and 0 is the number of currently matched base pairs. Procedure *bidir-search-stem* reports matching positions of $\mathcal{Q}$ whenever the boundaries of the RSSP are reached or $minbps < bpcount < maxbps$ holds.

References

[1] D. Strothmann. The affix array data structure and its applications to RNA secondary structure analysis. *Theor. Comput. Sci.*, 389(1-2):278–294, 2007.

[2] T. Schnattinger, E. Ohlebusch, and S. Gog. Bidirectional search in a string with wavelet trees. In *Proceedings of the 21st Annual Symposium on Combinatorial Pattern Matching*, volume 6129, pages 40–50. Springer, 2010.

[3] K. Darty, A. Denise, and Y. Ponty. VARNA: Interactive drawing and editing of the RNA seondary structure. *Bioinformatics*, 25(15):1974–1975, 2009.

Algorithm 3: *bidir-search-loop*(affix-interval $v = \langle k, \ell - [i..j], X\rangle$, pos r, pos r', *allowrightext*)

if $r' \leq maxloopend$ and $allowrightext$ = true **then**
  `// perform right extension`
  if $r' > loopend$ **then**
    chr' = 'N'
  else
    $chr' = P[r']$
  end
  foreach v' such that $d \in \varphi(chr')$ and $\overrightarrow{v}' = \overrightarrow{v}d$ **do**
    if $r < loopstart$ and $r' + 1 > loopend$ **then**
      if $msl = 0$ **then** `// if entire pattern is single stranded`
        report match at positions $\mathsf{suf}_X[i] + k, ..., \mathsf{suf}_X[j] + k$
        return
      else `// otherwise loop of length` $r' - r + 1$ `was matched`
        `// so extend stem region`
        bidir-search-stem(v', $loopstart - 1$, $loopend + 1$, 0)
      end
    end
    if $r' + 1 \leq maxloopend$ **then**
      bidir-search-loop(v', r, $r' + 1$, true)
    end
    if $r' + 1 > loopend$ **then**
      bidir-search-loop(v', r, $r' + 1$, false)
    end
  end
else if $r \geq minloopstart$ **then**
  `// perform left extension`
  if $r < loopstart$ **then**
    chr = 'N'
  else
    $chr = P[r]$
  end
  foreach v' such that $d \in \varphi(chr)$ and $\overrightarrow{v}' = d\overrightarrow{v}$ **do**
    if $r - 1 < loopstart$ and $r' > loopend$ **then**
      if $msl = 0$ **then** `// if entire pattern is single stranded`
        report match at positions $\mathsf{suf}_X[i] + k, ..., \mathsf{suf}_X[j] + k$
        return
      else `// otherwise loop of length` $r' - r + 1$ `was matched`
        `// so extend stem region`
        bidir-search-stem(v', $loopstart - 1$, $loopend + 1$, 0)
      end
    end
    bidir-search-loop(v', $r - 1$, r', *allowrightext*)
  end
end

Figure S1: Bidirectional recursive matching of the loop region of a variable-length RSSP using an affix array. Procedure *bidir-search-loop* searches for an RSSP (P, R) defined with additional variables *maxleftloopextent (mllex)* and *maxrightloopextent (mrlex)* denoting the maximum number of left and right extensions of the loop specified in R, respectively, and *maxstemlength (msl)* denoting the maximum number of base pairs. Used variables *loopstart*, *minloopstart*, *loopend*, and *maxloopend* are preset according to structure string R, *mllex*, and *mrlex* (see text). *bidir-search-loop* calls procedure *bidir-search-stem* (see Algorithm 4) whenever substrings of minimum length $loopend - loopstart + 1$ matching the loop are found.

Algorithm 4: *bidir-search-stem*(affix-interval $v = \langle k, \ell - [i..j], X \rangle$, pos r, pos r', *bpcount*)

```
if (r < 0 and r' ≥ m)  or  (minbps < bpcount < maxbps) then
    report match at positions suf_X[i] + k, ..., suf_X[j] + k
end
if (minbps < bpcount < maxbps  or  (r ≥ 0  and  r' < m  and  R[r] = '('  and  R[r'] = ')')
then
    if minbps < bpcount < maxbps then
        chr  = 'N'
        chr' = 'N'
    else
        chr  = P[r]
        chr' = P[r']
    end
    if X = R then
        // perform left extension first
        foreach v' such that d ∈ φ(chr) and →v' = d→v do
            foreach v'' such that e ∈ φ(chr') and (d, e) complementary and →v'' = →v'e do
                bidir-search-stem(v'', r − 1, r' + 1, bpcount + 1)
            end
        end
    else
        // perform right extension first
        foreach v' such that e ∈ φ(chr') and →v' = →ve do
            foreach v'' such that d ∈ φ(chr) and (d, e) complementary and →v'' = d→v' do
                bidir-search-stem(v'', r − 1, r' + 1, bpcount + 1)
            end
        end
    end
else if r' < m  and  R[r'] = '.' and (X = F or r < 0 or R[r] ≠ '.') then
    foreach v' such that d ∈ φ(P[r']) and →v' = →vd do
        bidir-search-stem(v', r, r' + 1, bpcount)
    end
else if r ≥ 0  and  R[r] = '.' then
    foreach v' such that d ∈ φ(P[r]) and →v' = d→v do
        bidir-search-stem(v', r − 1, r', bpcount)
    end
end
```

Figure S2: Bidirectional recursive matching of the stem region of a variable-length RSSP using an affix array. Procedure *bidir-search-stem* is called by procedure *bidir-search-loop* (see Algorithm 3) and extends substrings $\overrightarrow{v}$ matching the loop region of the RSSP (P, R) to substrings matching also the stem. Used variables *minbps* and *maxbps* are preset according to structure string R and variable *maxstemlength (msl)* (see text).

Organism	Genome size (n)	$\mathsf{suf_F}$ (4n)	$\mathsf{lcp_F}$ (n)	$\mathsf{lcpe_F}$	$\mathsf{aflk_F}$ (4n)	$\mathsf{suf_R}$ (4n)	$\mathsf{lcp_R}$ (n)	$\mathsf{lcpe_R}$	$\mathsf{aflk_R}$ (4n)
C.elegans	100.29	401.14	100.29	6.29	401.14	401.14	100.29	6.29	401.14
A.thaliana	119.67	478.67	119.67	8.85	478.67	478.67	119.67	8.85	478.67
D.melanogaster	168.74	674.95	168.74	94.34	674.95	674.95	168.74	94.34	674.95
C.intestinalis	173.52	694.02	173.50	28.03	694.02	694.02	173.50	28.03	694.02
O.sativa	374.33	1,497.33	374.33	71.05	1,497.33	1,497.33	374.33	71.05	1,497.33
M.gallopavo	1,087.50	4,349.99	1,087.50	2.01	4,349.99	4,349.99	1,087.50	2.01	4,349.99
G.gallus	1,108.48	4,433.93	1,108.48	98.86	4,433.93	4,433.93	1,108.48	98.86	4,433.93
D.rerio	1,481.32	5,925.08	1,481.27	457.26	5,925.08	5,925.08	1,481.27	457.26	5,925.08
X.tropicalis	1,510.98	6,043.63	1,510.91	310.89	6,043.63	6,043.63	1,510.91	310.89	6,043.63
P.vampyrus	1,999.71	7,998.82	1,999.71	170.84	7,998.82	7,998.82	1,999.71	170.84	7,998.82

Table S2: Sizes in megabytes of the different tables the affix array consists of for the genomes used in Experiment 1. $\mathsf{lcpe_F}$ and $\mathsf{lcpe_R}$ are the exception tables storing entries with value larger than 255 which cannot be stored in tables $\mathsf{lcp_F}$ and $\mathsf{lcp_R}$, respectively. In tables $\mathsf{lcpe_F}$ and $\mathsf{lcpe_R}$, each entry consumes 8 bytes.

Organism	Genome size	BWI
C.elegans	100.29	157.96
A.thaliana	119.67	188.59
D.melanogaster	168.74	295.37
C.intestinalis	173.52	279.83
O.sativa	374.33	602.21
M.gallopavo	1,087.50	1,800.88
G.gallus	1,108.48	1,757.84
D.rerio	1,481.32	2,424.81
X.tropicalis	1,510.98	2,309.24
P.vampyrus	1,999.71	3,282.55

Table S3: Size in megabytes of the bidirectional wavelet index (BWI) [2] for different genomes.

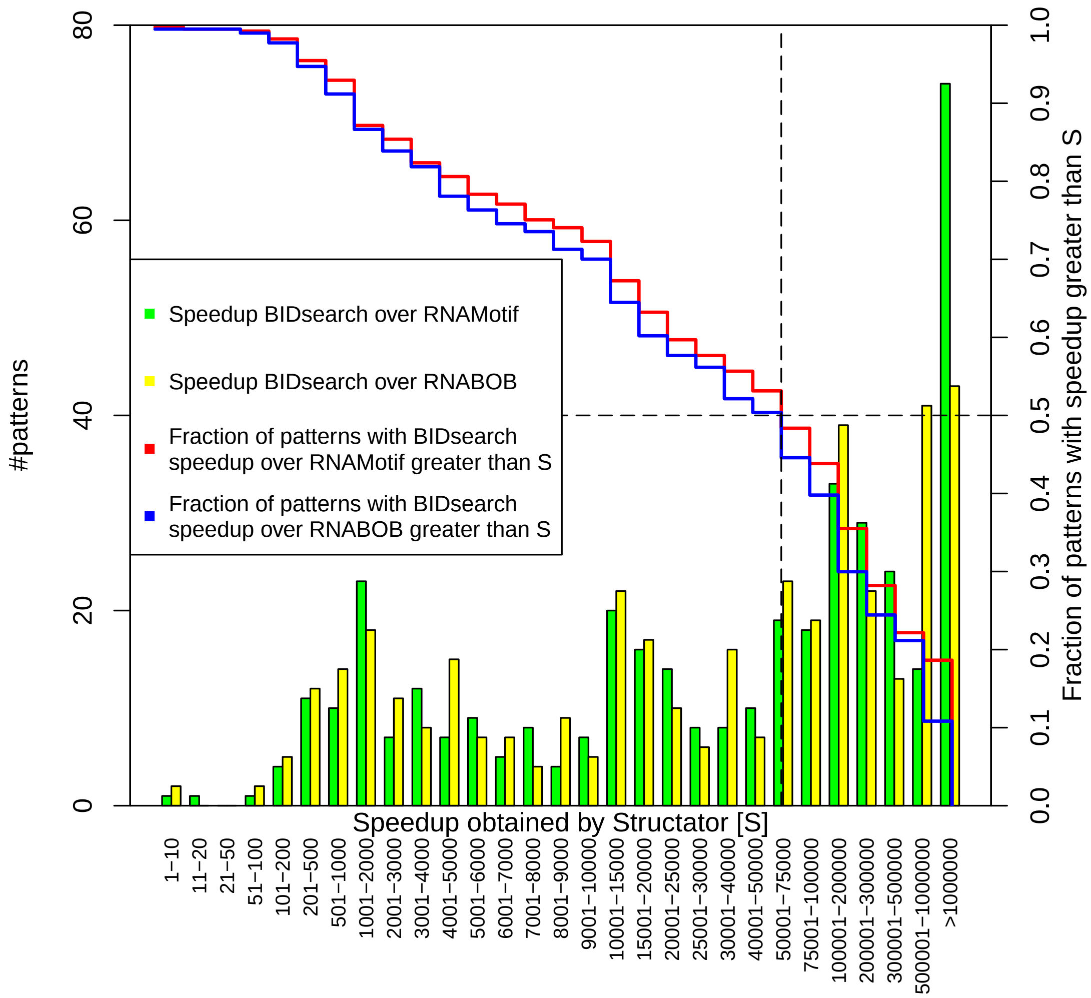

Figure S3: Distribution of speedup factors of *BIDsearch* over *RNABOB* (yellow) and *RNAMotif* (green) when searching for 397 RSSPs in RFAM10 consisting of ~ 622 megabases of RNA sequence data. The red and blue curves show the values of one minus the empirical cumulative distribution function of the speedup factors distributions. That is, for a given speedup factor S they show the fraction of RSSPs for which *BIDsearch* obtained a speedup greater than S over *RNAMotif* (red curve) and *RNABOB* (blue curve), respectively. We observed that *BIDsearch* is more than 50,000 times faster than *RNABOB* and *RNAMotif* for the majority of the patterns (see intersection point of dashed lines). Moreover, the total search time required by *BIDsearch* is dominated by only a small number of patterns describing large unconserved loop regions.

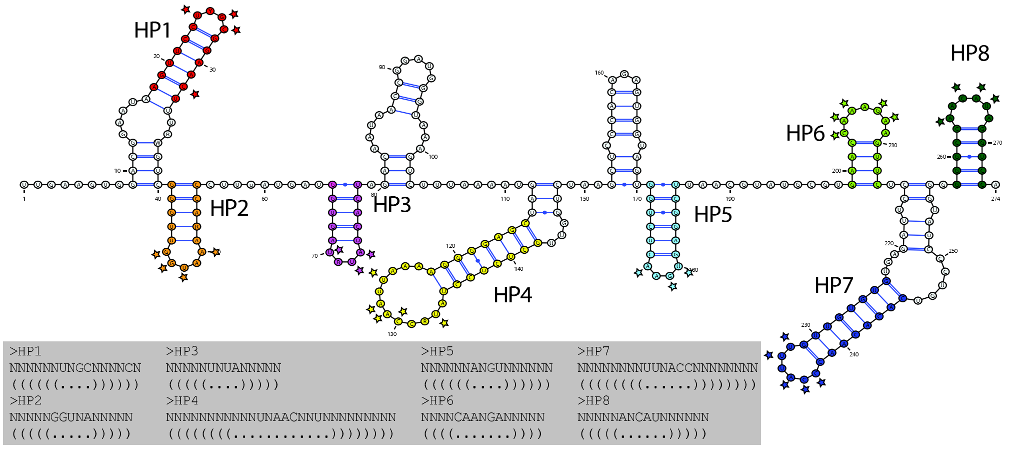


```
>HP1                >HP3                              >HP5                 >HP7
NNNNNNUNGCNNNNCN    NNNNNUNUANNNNN                    NNNNNNANGUNNNNNN     NNNNNNNNUUNACCNNNNNNNN
((((((....))))))    (((((....)))))                    ((((((....))))))     ((((((((......))))))))
>HP2                >HP4                              >HP6                 >HP8
NNNNNGGUNANNNNN     NNNNNNNNNNNUNAACNNUNNNNNNNNN      NNNNCAANGANNNNN      NNNNNANCAUNNNNNN
(((((.....)))))     ((((((((...........))))))))       ((((.......))))      (((((......)))))
```

Figure S4: Consensus secondary structure of the CTV_rep_sig family (RFAM Acc.: RF00193) visualized with the *VARNA* program [3] and SSD in *Structator* syntax describing this family. The 8 given RSSPs correspond to the colored stem-loops HP1 - HP8. Positions at which sequence information is used in the descriptor are marked with an asterisk.

Acc.	#Matching chains	#TP	#FP	#FN	Sensitivity	Specificity	Accuracy	Precision	#RSSPs	Min.chain length	$T_{BIDsearch}$[sec]	$T_{ONLsearch}$[sec]	Speedup	$T_{chaining}$[sec]
RF00044	8	8	0	0	1.000	1.000	1.000	1.000	8	2	0.964	117.359	121.742	0.001
RF00193	37	37	0	0	1.000	1.000	1.000	1.000	8	5	1.220	140.681	115.312	0.063
RF00126	106	106	0	1	0.991	1.000	1.000	1.000	6	2	1.032	128.476	124.492	0.000
RF00503	78	78	0	2	0.975	1.000	1.000	1.000	10	2	1.084	164.866	152.090	0.002
RF00209	1,511	1,493	18	58	0.963	1.000	1.000	0.988	9	2	1.056	129.372	122.511	0.006
RF00625	24	22	2	1	0.957	1.000	1.000	0.917	5	3	3.304	102.066	30.892	0.656
RF00061	6,211	6,211	0	285	0.956	1.000	1.000	1.000	7	4	1.188	119.239	100.370	0.032
RF00224	21	21	0	1	0.955	1.000	1.000	1.000	10	3	1.508	202.661	134.391	0.138
RF00084	111	111	0	7	0.941	1.000	1.000	1.000	4	2	1.180	78.669	66.669	0.050
RF00372	42	42	0	3	0.933	1.000	1.000	1.000	7	3	1.092	104.663	95.845	0.007
RF00115	58	58	0	5	0.921	1.000	1.000	1.000	9	4	1.128	167.962	148.902	0.024
RF00488	24	24	0	3	0.889	1.000	1.000	1.000	6	4	1.084	94.938	87.581	0.043
RF00294	44	44	0	9	0.830	1.000	1.000	1.000	12	3	1.124	164.814	146.632	0.008
RF00210	345	345	0	72	0.827	1.000	1.000	1.000	14	3	1.308	206.133	157.594	0.104
RF00228	348	346	2	79	0.814	1.000	1.000	0.994	13	2	1.048	225.982	215.632	0.006
RF00036	18,312	18,312	0	4,452	0.804	1.000	0.999	1.000	16	3	1.464	224.778	153.537	0.145
RF00549	39	38	1	10	0.792	1.000	1.000	0.974	10	4	1.584	154.382	97.463	0.142
RF00448	11	11	0	3	0.786	1.000	1.000	1.000	7	4	1.000	102.730	102.730	0.002
RF00177	584,748	582,839	1,909	179,250	0.765	0.999	0.946	0.997	13	3	11.004	221.798	20.156	2.414
RF00101	142	142	0	45	0.759	1.000	1.000	1.000	6	3	1.000	119.407	119.407	0.004
RF00166	54	54	0	18	0.750	1.000	1.000	1.000	8	3	1.068	127.872	119.730	0.009
RF00018	278	272	6	96	0.739	1.000	1.000	0.978	11	5	3.944	212.133	53.786	0.666
RF00252	26	26	0	10	0.722	1.000	1.000	1.000	10	3	1.260	143.709	114.055	0.057
RF00547	39	39	0	18	0.684	1.000	1.000	1.000	14	3	2.604	221.458	85.045	0.452
RF00011	355	353	2	185	0.656	1.000	1.000	0.994	10	4	2.988	183.923	61.554	0.582
RF00010	2,478	2,402	76	1,679	0.589	1.000	0.999	0.969	12	5	6.212	187.616	30.202	1.548
RF00449	33	32	1	26	0.552	1.000	1.000	0.970	9	3	1.308	154.726	118.292	0.073
RF00040	92	92	0	82	0.529	1.000	1.000	1.000	9	4	1.248	153.410	122.925	0.050
RF00023	1,362	1,362	0	1,699	0.445	1.000	0.999	1.000	11	3	2.076	193.740	93.324	0.229
RF00229	1,257	1,256	1	1,637	0.434	1.000	0.999	0.999	11	3	1.472	193.168	131.228	0.139
RF00222	26	26	0	35	0.426	1.000	1.000	1.000	12	3	1.148	201.557	175.572	0.025
RF00459	223	215	8	341	0.387	1.000	1.000	0.964	7	2	4.776	221.002	46.273	0.012
RF00028	10,647	10,229	418	28,820	0.262	1.000	0.991	0.961	13	2	1.476	203.889	138.136	0.075
RF00261	21	21	0	65	0.244	1.000	1.000	1.000	8	4	1.552	171.063	110.221	0.130
RF00373	82	75	7	247	0.233	1.000	1.000	0.915	8	4	1.692	143.645	84.897	0.166
RF00230	2,059	1,753	306	6,507	0.212	1.000	0.998	0.851	8	3	39.006	220.410	5.651	0.471
RF00226	18	18	0	73	0.198	1.000	1.000	1.000	7	4	2.664	108.687	40.798	0.449
RF00009	136	111	25	455	0.196	1.000	1.000	0.816	11	3	3.260	190.164	58.333	0.480
RF00629	6	6	0	25	0.194	1.000	1.000	1.000	8	4	1.816	153.526	84.541	0.248
RF00030	20	20	0	476	0.040	1.000	1.000	1.000	9	5	10.632	175.559	16.512	2.427
RF00100	614	614	0	15,042	0.039	1.000	0.995	1.000	13	7	1.240	198.652	160.203	0.065
RF00004	257	257	0	7,252	0.034	1.000	0.998	1.000	8	4	1.320	128.812	97.585	0.034
**Average(∅):**					**0.629**	**1.000**	**0.998**	**0.983**	**9.45**	**3.38**	**3.100**	**163.330**	**101.500**	**0.29**
**Total(Σ):**									**397**		**130.13**	**6,859.7**		**12.236**

Table S4: Results of *Structator* searches on RFAM10 (1,446 families; 3,192,599 sequences) using SSDs describing 42 Rfam families. The manually compiled SSDs used in this experiment are available on the *Structator* website. They were designed to be highly specific and consist of 397 RSSPs in total with an average of 9.45 RSSPs per SSD. These are the same 397 RSSPs used in section "Searching large sequence databases" in the main document. Columns 2, 3, 4, and 5 show the number of sequences containing high-scoring global chains, the numbers of true positives (TP), false positives (FP), and false negatives (FN), respectively. Sensitivity is computed as $\frac{\#TP}{\#TP+\#FN}$, specificity as $\frac{\#TN}{\#TN+\#FP}$, accuracy as $\frac{\#TP+\#TN}{\#TP+\#FP+\#FN+\#TN}$, and precision as $\frac{\#TP}{\#TP+\#FP}$. Observe that these values strongly depend on the used SSD. The number of RSSPs constituting an SSD is given in column 10. Column 11 shows the minimal required length of a chain to be considered a matching chain. Total running times of *Structator* operating in *BIDsearch* and *ONLsearch* mode are given in columns 12 and 13, respectively. Column 14 shows *BIDsearch*'s speedups over *ONLsearch*. The running time required for chaining of RSSP matches is listed in column 15. Observe that the sum of running times does not match the times needed for searching with the 397 single RSSPs reported in the main document because here each SSD was searched using a separate *Structator* program call.

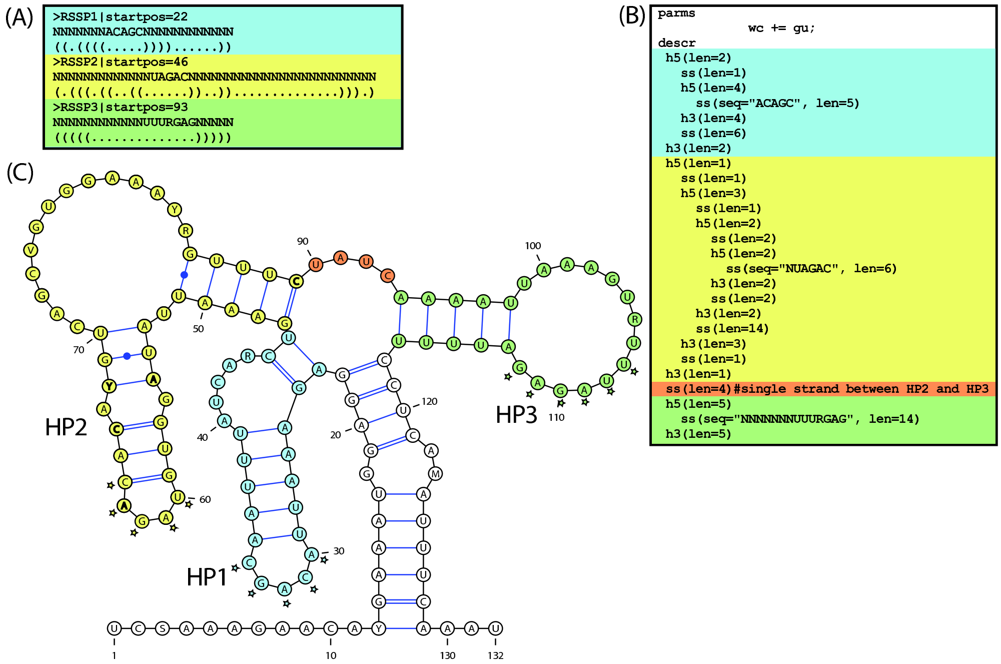

Figure S5: (A) SSD for HAR1F RNA family consisting of RSSP1, RSSP2, and RSSP3 in *Structator* syntax. RSSPs were built from stem-loops HP1, HP2, and HP3 shown in (C). (B) *RNAMotif* descriptor for the same structural elements. Secondary structure drawing shown in (C) was generated with *VARNA* [3].
